# Supplementary figures and images for: Natural arbovirus infection rate and detectability of indoor female Aedes aegypti from Mérida, Yucatán, Mexico
Source: PLoS Negl Trop Dis. 2021 Jan 4;15(1):e0008972. doi: 10.1371/journal.pntd.0008972 (PMC7781390; doi:10.1371/journal.pntd.0008972)

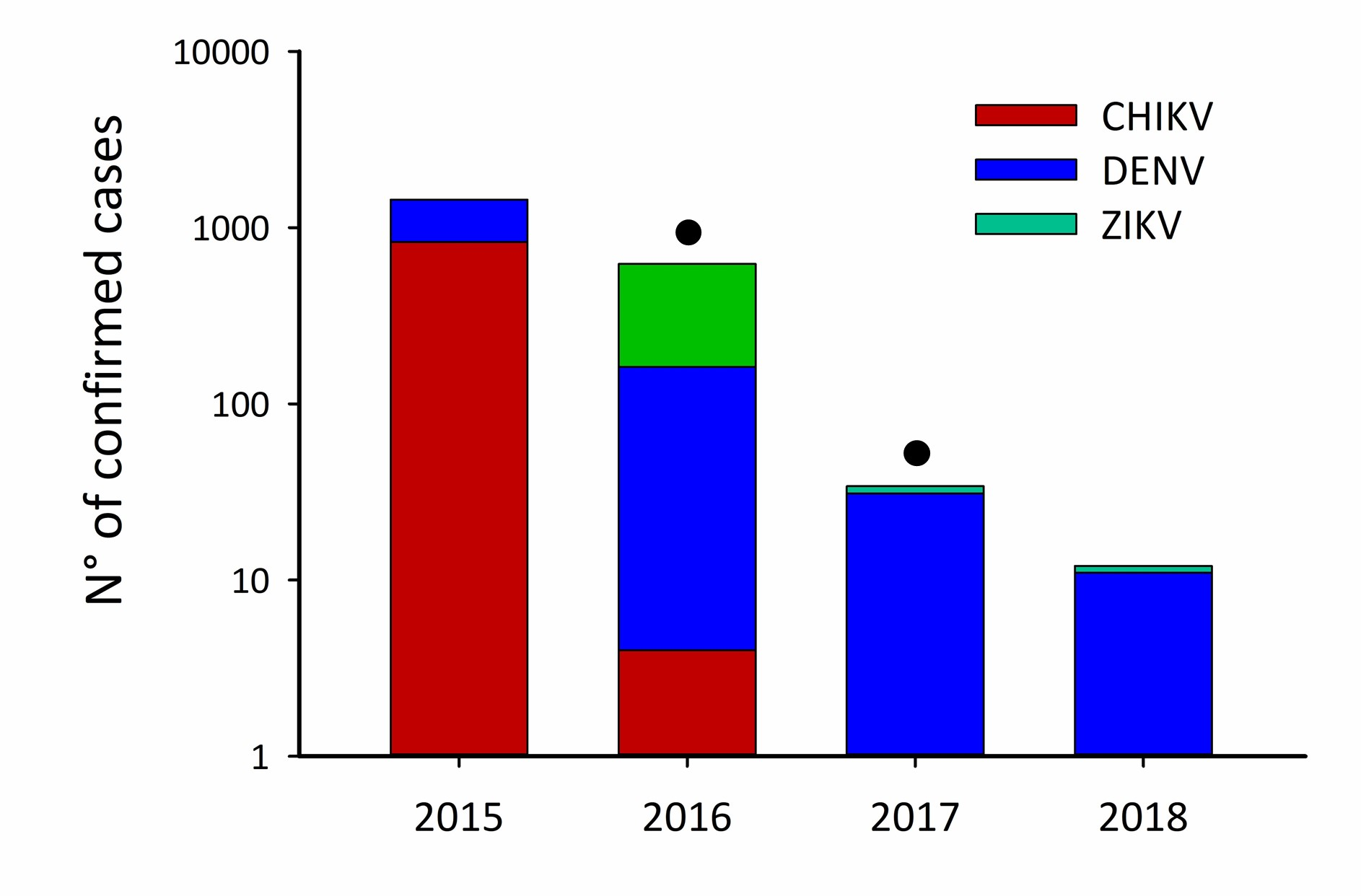

Supplement: S1 Fig — Mexico. Data was obtained from SINAVE database, number of cases caused by CHIKV in 2015 was obtained from Méndez et al. 2017 [74]. Axis Y (Number of confirmed cases) is presented in Logarithmic scale. Dots on top of each bar represent the year of mosquito collection. (TIF) [file pntd.0008972.s001.tif]

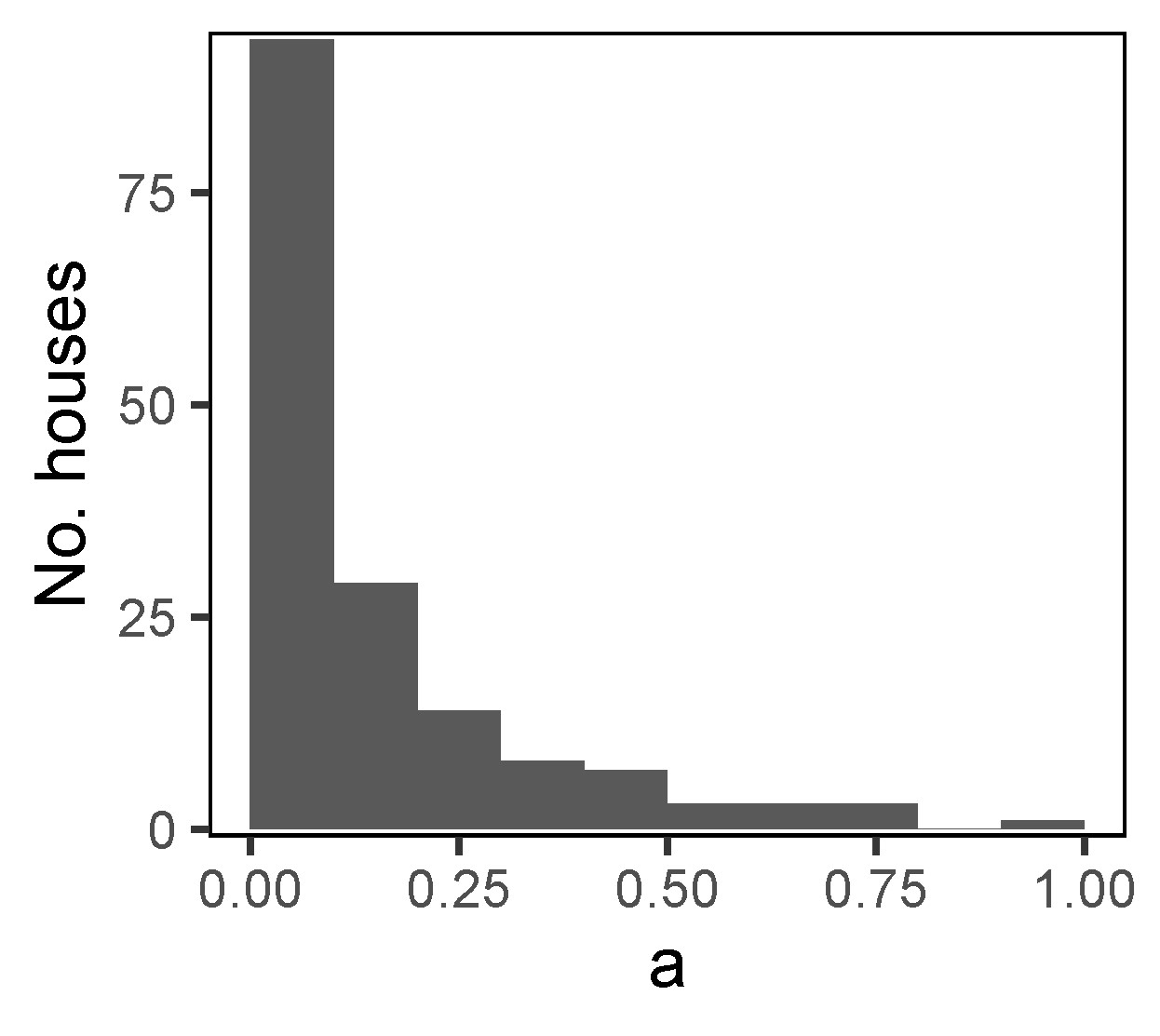

Supplement: S2 Fig — Distribution of human biting rate (a) by house. (TIF) [file pntd.0008972.s002.tif]
