# Supplementary material for: Natural arbovirus infection rate and detectability of indoor female Aedes aegypti from Mérida, Yucatán, Mexico
Source: PLoS Negl Trop Dis. 2021 Jan 4;15(1):e0008972. doi: 10.1371/journal.pntd.0008972 (PMC7781390; doi:10.1371/journal.pntd.0008972)
Supplement: S1 Table — (DOCX) [file pntd.0008972.s003.docx]

| **Primer** | **Sequence** | **Probe** | **Working**  **Concentration (µM)** |
| --- | --- | --- | --- |
| panDENf | AAGGACTAGAGGTTAKAGGAGACCC | Quasar670 (Cy5)-BHQ2 | 25 |
| panDENr | CGYTCTGTGCCTGGAWTGATG |  | 25 |
| panDENp | AACAGCATATTGACGCTGGGAIAGACCAG |  | 10 |
| CHIKf6856 | TCACTCCCTGTTGGACTTGATAGA | TAMRA-BHQ2 | 25 |
| CHIKr 6981 | TTGACGAACAGAGTTAGGAACATACC |  | 25 |
| CHIKp 6919 | AGGTACGCGCTTCAAGTTCGGCG |  | 10 |
| Zika1087f | CCGCTGCCCAACACAAG | FAM-BHQ1 | 25 |
| Zika1163cr | CCACTAACGTTCTTTTGCAGACAT |  | 25 |
| Zika1108p | AGCCTACCTTGACAAGCAGTCAGACACTCAA |  | 10 |
