# Supplementary material for: Natural arbovirus infection rate and detectability of indoor female Aedes aegypti from Mérida, Yucatán, Mexico
Source: PLoS Negl Trop Dis. 2021 Jan 4;15(1):e0008972. doi: 10.1371/journal.pntd.0008972 (PMC7781390; doi:10.1371/journal.pntd.0008972)
Supplement: S2 Table — (DOCX) [file pntd.0008972.s004.docx]

|  | **Infection in Bodies** | | | **Infection in Heads** | | |
| --- | --- | --- | --- | --- | --- | --- |
|  | **OR** | **2.5%** | **97.5%** | **OR** | **2.50%** | **97.50%** |
| **Sella 1** | 0.0 | 0.0 | 0.0 | 0.0 | 0.0 | 0.0 |
| **Sella 2** | 1.3 | 0.7 | 2.5 | 1.5 | 0.5 | 4.4 |
| **Sella 3** | 2.2 | 1.0 | 5.4 | 1.6 | 0.4 | 6.4 |
| **Sella 4** | 1.1 | 0.5 | 2.5 | 0.8 | 1.0 | 3.7 |
| **Sella 5** | 1.3 | 0.5 | 3.5 | 0.6 | 0.1 | 6.1 |
| **Sella 6** | 1.0 | 0.4 | 2.4 | 1.2 | 0.3 | 4.9 |
| **Sella 7** | 0.8 | 0.9 | 1.8 | 0.7 | 0.9 | 2.5 |
