# Supplementary material for: Natural arbovirus infection rate and detectability of indoor female Aedes aegypti from Mérida, Yucatán, Mexico
Source: PLoS Negl Trop Dis. 2021 Jan 4;15(1):e0008972. doi: 10.1371/journal.pntd.0008972 (PMC7781390; doi:10.1371/journal.pntd.0008972)
Supplement: S3 Table — (DOCX) [file pntd.0008972.s005.docx]

| Model* | Parameter | Estimate | SE | t | P |
| --- | --- | --- | --- | --- | --- |
| EIR~Total catch | Intercept | 0.0379 | 0.0232 | 1.629 | 0.106 |
|  | Total catch | 0.0020 | 0.0009 | 2.181 | 0.031 |
| VC~Total catch | Intercept | -0.3323 | 0.1050 | -3.166 | 0.001 |
|  | Total catch | 0.0744 | 0.0046 | 16.155 | < 2e-16 |
| *Models presented were selected using multi-model selection, based on AIC. | | | | | |
