# Supplementary material for: Natural arbovirus infection rate and detectability of indoor female Aedes aegypti from Mérida, Yucatán, Mexico
Source: PLoS Negl Trop Dis. 2021 Jan 4;15(1):e0008972. doi: 10.1371/journal.pntd.0008972 (PMC7781390; doi:10.1371/journal.pntd.0008972)
Supplement: S4 Table — (DOCX) [file pntd.0008972.s006.docx]

| **Virus** | **Samples Pos** | **Seq Samples** | **Blast Samples** | |
| --- | --- | --- | --- | --- |
| CHIKV | 129 | 100 | 92 | 76 Consensus |
|  |  |  |  | 16 Singlets |
| DENV^ | 19 | 17 | 12 | 3 Consensus |
|  |  |  |  | 9 Singlets post t |
| ZIKV | 18 | 17 | 17 | 7 Consensus |
|  |  |  |  | 10 Singlets |
|  | --> | 7 heads* | 7 | 5 Consensus |
|  |  |  |  | 2 Singlets |
|  | --> | 3 co-CHIKV | 3 | 3 Consensus |

* ZIKV heads matches with positive bodies.

^ DENV serotype corresponded to DENV-4
