# Supplementary material for: Natural arbovirus infection rate and detectability of indoor female Aedes aegypti from Mérida, Yucatán, Mexico
Source: PLoS Negl Trop Dis. 2021 Jan 4;15(1):e0008972. doi: 10.1371/journal.pntd.0008972 (PMC7781390; doi:10.1371/journal.pntd.0008972)

PLOS Neglected Tropical Diseases

Natural Arbovirus Infection Rate and Detectability of Indoor Female Aedes aegypti from Merida, Yucatan, Mexico.

--Manuscript Draft--

**Manuscript Number:**	PNTD-D-20-01322
**Full Title:**	Natural Arbovirus Infection Rate and Detectability of Indoor Female Aedes aegypti from Merida, Yucatan, Mexico.
**Short Title:**	Natural Arbovirus Infection Rate and Detectability of Indoor Female Aedes aegypti.
**Article Type:**	Research Article
**Keywords:**	Sampling, Prokopack, Aedes, Abundance, Density, Infection, Arbovirus
**Abstract:**	Background: As Aedes -borne viruses (ABV) continue their resurgence and global expansion, there is a need to better quantify virus presence, co-circulation and transmission risk. Arbovirus infection in Aedes aegypti has historically been quantified over a small fraction of the adult population by pooling collected mosquitoes to increase detectability. However, there is a significant knowledge gap about the magnitude of natural arbovirus infection within areas of active transmission, as well as the sensitivity of detection of such an approach. Here we quantify the absolute Aedes aegypti density in houses with suspected active virus transmission, the absolute arbovirus infection rate in individually tested Aedes aegypti females, the sensitivity of using Prokopack aspirators in detecting arbovirus infected mosquitoes, and entomological inoculation rate (EIR) and vectorial capacity (VC) two measures ABV transmission potential.<br>Methodology/Principal Findings : We individually tested by RT-PCR 2,161 Aedes aegypti females collected indoors from 200 houses using Prokopack aspirators and found that 7.7% of them were positive to any ABV. Most infections were CHIKV (77.7%), followed by DENV (11.4%) and ZIKV (9.0%). The distribution of infected Aedes aegypti was overdispersed, 33% of houses contributing with 81% of the infected mosquitoes. A significant association between ABV infection and Aedes aegypti density indoors was found when the total catch, rather than number sampled, of Aedes aegypti was considered. Indeed, such lack of association was driven by a low sensitivity of routine indoor aspirator collections in detecting ABV infections (sensitivity was 16.3% and 23.4% for detecting individually infected mosquitoes and houses, respectively). When averaged across all infested houses, mean EIR ranged between 0.04 and 0.06 infective bites per person per day, and mean VC was 0.6 infectious vectors generated from a population feeding on a single infected host per house/day. Both measures were significantly and positively associated with total density indoors.<br>Conclusions/Significance: Our findings provide evidence that the accurate estimation and quantification of arbovirus infection rate and transmission risk is a function of the collection method used, the sampling effort, the local abundance of Aedes aegypti and the intensity of arbovirus circulation.
**Additional Information:**	
**Question**	**Response**
**Financial Disclosure**<br><br>Enter a financial disclosure statement that describes the sources of funding for the work included in this submission. Review the submission guidelines for detailed requirements. View published research articles from *PLOS NTDs* for specific examples.	The funders had no role in study design, data collection and analysis, decision to publish, or preparation of the manuscript.

Powered by Editorial Manager® and ProduXion Manager® from Aries Systems Corporation

This statement is required for submission and **will appear in the published article** if the submission is accepted. Please make sure it is accurate.<br><br>**Unfunded studies**<br><br>Enter: *The author(s) received no specific funding for this work.*<br><br>**Funded studies**<br><br>Enter a statement with the following details:<br>• Initials of the authors who received each award<br>• Grant numbers awarded to each author<br>• The full name of each funder<br>• URL of each funder website<br>• Did the sponsors or funders play any role in the study design, data collection and analysis, decision to publish, or preparation of the manuscript?<br>• **NO** - Include this sentence at the end of your statement: *The funders had no role in study design, data collection and analysis, decision to publish, or preparation of the manuscript.*<br>• **YES** - Specify the role(s) played.<br><br>* typeset	
**Competing Interests**<br><br>Use the instructions below to enter a competing interest statement for this submission. On behalf of all authors, disclose any competing interests that could be perceived to bias this work—acknowledging all financial support and any other relevant financial or non-financial competing interests.<br><br>This statement **will appear in the published article** if the submission is	The authors have declared that no competing interests exist.

accepted. Please make sure it is accurate. View published research articles from *PLOS NTDs* for specific examples.<br><br>**NO authors have competing interests**<br><br>Enter: *The authors have declared that no competing interests exist.*<br><br>**Authors with competing interests**<br><br>Enter competing interest details beginning with this statement:<br><br>*I have read the journal's policy and the authors of this manuscript have the following competing interests: [insert competing interests here]*<br><br>* typeset	
**Data Availability**<br><br>Authors are required to make all data underlying the findings described fully available, without restriction, and from the time of publication. PLOS allows rare exceptions to address legal and ethical concerns. See the PLOS Data Policy and FAQ for detailed information.<br><br>A Data Availability Statement describing where the data can be found is required at submission. Your answers to this question constitute the Data Availability Statement and **will be published in the article**, if accepted.	Yes - all data are fully available without restriction

**Important:** Stating 'data available on request from the author' is not sufficient. If your data are only available upon request, select 'No' for the first question and explain your exceptional situation in the text box.<br><br>Do the authors confirm that all data underlying the findings described in their manuscript are fully available without restriction?	
**Describe where the data may be found in full sentences. If you are copying our sample text, replace any instances of XXX with the appropriate details.**<br><br>• If the data are **held or will be held in a public repository**, include URLs, accession numbers or DOIs. If this information will only be available after acceptance, indicate this by ticking the box below. For example: *All XXX files are available from the XXX database (accession number(s) XXX, XXX.*).<br>• If the data are all contained **within the manuscript and/or Supporting Information files**, enter the following: *All relevant data are within the manuscript and its Supporting Information files.*<br>• If neither of these applies but you are able to provide **details of access elsewhere**, with or without limitations, please do so. For example:<br><br>*Data cannot be shared publicly because of [XXX]. Data are available from the XXX Institutional Data Access / Ethics Committee (contact via XXX) for researchers who meet the criteria for access to confidential data.*	The data underlying the results presented in the study are available from the corresponding author upon request.

The data underlying the results presented in the study are available from (include the name of the third party and contact information or URL).<br>• This text is appropriate if the data are owned by a third party and authors do not have permission to share the data.<br><br>* typeset	
Additional data availability information:	Tick here if the URLs/accession numbers/DOIs will be available only after acceptance of the manuscript for publication so that we can ensure their inclusion before publication.

Powered by Editorial Manager® and ProduXion Manager® from Aries Systems Corporation

Manuscript

Click here to access/download;Manuscript;Kirstein_etal.2020_Manuscript.pdf

Natural Arbovirus Infection Rate and Detectability of Indoor Female *Aedes aegypti* from Merida, Yucatan, Mexico.

Oscar David Kirstein[1, ¶], Guadalupe Ayora-Talavera[3, ¶], Edgar Koyoc-Cardeña[2], Daniel Chan Espinoza[2], Azael Che-Mendoza[2], Azael Cohuo-Rodriguez[2], Pilar Granja-Pérez[5], Henry Puerta-Guardo[2], Norma Pavia-Ruz[4], Mike W. Dunbar[1], Pablo Manrique-Saide[2] and Gonzalo M. Vazquez-Prokopec[1*]

[1]Department of Environmental Sciences, Emory University, Atlanta, GA, USA.

[2]Unidad Colaborativa de Bioensayos Entomológicos, Campus de Ciencias Biológicas y Agropecuarias, Universidad Autónoma de Yucatán, Mérida, Yucatán, México.

[3]Laboratorio de Virología. Centro de Investigaciones Regionales “Dr. Hideyo Noguchi”, Universidad Autónoma de Yucatán, Mérida, Yucatán, México.

[4]Laboratorio de Hematología. Centro de Investigaciones Regionales “Dr. Hideyo Noguchi”, Universidad Autónoma de Yucatán, Mérida, Yucatán, México.

[5]Laboratorio Estatal de Salud Pública, Servicios de Salud de Yucatán, Mérida, Yucatán, México.

[¶] These authors contributed equally to this work.

[*]Correspondence: gmvazqu@emory.edu

Abstract

Background

As *Aedes*-borne viruses (ABV) continue their resurgence and global expansion, there is a need to better quantify virus presence, co-circulation and transmission risk. Arbovirus infection in *Aedes aegypti* has historically been quantified over a sample of the adult population by pooling collected mosquitoes to increase detectability. However, there is a significant knowledge gap about the magnitude of natural arbovirus infection within areas of active transmission, as well as the sensitivity of detection of such an approach. Here we quantify the absolute *Aedes aegypti* density in houses with suspected active virus transmission, the absolute arbovirus infection rate in individually tested *Aedes aegypti* females, the sensitivity of using Prokopack aspirators in detecting arbovirus infected mosquitoes, and entomological inoculation rate (EIR) and vectorial capacity (VC), two measures ABV transmission potential.

Methodology/Principal Findings

We individually tested by RT-PCR 2,161 *Aedes aegypti* females collected indoors from 200 houses using Prokopack aspirators and found that 7.7% of them were positive to any ABV. Most infections were CHIKV (77.7%), followed by DENV (11.4%) and ZIKV (9.0%). The distribution of infected *Aedes aegypti* was overdispersed, 33% of houses contributed with 81% of the infected mosquitoes. A significant association between ABV infection and *Aedes aegypti* density indoors was found when Ae. aegypti total catch, rather than number sampled, was considered. Indeed, such lack of association was driven by a low sensitivity of routine indoor aspirator collections in detecting ABV infections (sensitivity was 16.3% and 23.4% for detecting individually infected mosquitoes and houses, respectively). When averaged across all infested houses, mean EIR ranged between 0.04 and 0.06 infective bites per person per day, and mean VC was 0.6 infectious vectors generated from a population feeding on a single infected host per house/day. Both measures were significantly and positively associated with total *Ae. aegypti* density indoors.

Conclusions/Significance

Our findings provide evidence that the accurate estimation and quantification of arbovirus infection rate and transmission risk is a function of the collection method used, the sampling effort, the local abundance of *Aedes aegypti* and the intensity of arbovirus circulation.

Keywords: sampling, Prokopack, *Aedes*, abundance, density, infection, arbovirus

Author summary

Aedes-borne diseases comprise a serious public health burden in many parts of the world, usually affecting low income areas. The ability to detect virus circulation within a population may be key in responding to the threat of outbreaks, providing a cost-effective approach for triggering vector control. Unfortunately, gaps in the knowledge of natural *Aedes*-borne virus (ABV) infection in *Aedes aegypti* have led to uncertainties in the consideration of entomo-virological approaches for virus surveillance. Here, we show that the natural infection rate in a mosquito population may not be a function of where *Aedes aegypti* are, but rather where key human-mosquito contacts occur. Sampling 200 houses with suspected ABV active transmission led us to quantify high virus infection rates in all *Aedes aegypti* present in the house and use such information to estimate the sensitivity of indoor aspiration with Prokopack devices and two measures of ABV transmission potential. Our findings provide evidence that the accurate estimation and quantification of arbovirus infection rate and transmission risk is a function of the collection method used, the sampling effort, the local abundance of *Aedes aegypti* and the intensity of arbovirus circulation. Results from this study are relevant to understand the value of vector surveillance via entomo-virologic surveys, and for the design of entomological endpoints relevant for epidemiological trials quantifying the impact of vector control on ABVs.

Introduction

Emerging *Aedes*-borne viruses (ABVs) such as chikungunya (CHIKV), Dengue (DENV) and Zika (ZIKV) contribute significantly to the global burden of infectious diseases [1-3]. Transmitted primarily by the ubiquitous and highly anthropophilic mosquito *Aedes aegypti*, these viruses have propagated throughout tropical and subtropical urban environments often co-circulating within the same period and geographical areas [4-8]. Infections of CHIKV, DENV and ZIKV can present similar manifestation, ranging from asymptomatic to mild or inapparent to severe illness with life-threatening manifestations and death [6, 9]. ZIKV and CHIKV infections, particularly in the Americas, have been linked to fetus abnormalities during pregnancy, neurological complications, and chronic joint diseases in adults that can persist for even years [10, 11]. The co-circulation of arboviral infections and their epidemic propagation challenge differential diagnoses, primary patient care, and limit the effectiveness of existing vector control tools [5, 8, 12-15]. Furthermore, the lack of accurate entomological correlates of ABV risk [2, 16, 17], is affected by multiple sources of bias including the difficulty of detecting and accurately quantifying immature or adult *Ae. aegypti* density [18], the exposure of people to mosquitoes in residences other than their homes [19, 20], the variable level of susceptibility in the human population against each virus [21], or the limit of identification of entomological triggers for informing vector control [22].

Aedes aegypti is considered a very efficient vector of ABVs even at low apparent population densities [23, 24]. A common assumption in ABV research is that due to the low vector density and focal nature of human-mosquito contacts [19], natural arbovirus infection in *Ae. aegypti* is very low [25], limiting the implementation of entomo-virological surveillance systems as conducted for other urban arbovirus (e.g., West Nile virus [26]).

The estimation of infection rates in mosquito populations depends on the methodology used to detect viral infection. Methods for virus detection include cell culture [27, 28], immunoassay [27, 29] or molecular methods, with reverse transcription–polymerase chain reaction (RT-PCR) followed by

amplicon sequence [5, 8, 30]. The latter is considered the benchmark for infection confirmation and virus discrimination. Given their cost, and often limited mosquito yields, ABV detection tends to be conducted in pools of mosquitoes, generally between 10 and 20 individuals per pool [26]. In the presence of focal transmission (e.g., multiple infected mosquitoes within a single premise, infecting many individuals), such pooling method may lead to bias in the estimation of ABV natural infection rates [31, 32]. Part of this bias is introduced by the calculation of the minimum infection rates (MIR) and the maximum likelihood rate (MLR), which make different assumptions about the frequency and aggregation of infection rates, but that are not sensitive to extreme variability in the distribution of infected mosquitoes [26, 32, 33].

Despite these assumption and limitations, multiple research groups have quantified infection rates in *Ae. aegypti* with different levels of success. ABV entomo-virological characterization in *Ae. aegypti* from northern Brazil detected only 7 out of 37 pools (containing 10 mosquitoes each) tested and ~1000 mosquitoes collected [8]. A study conducted during the DENV transmission peak in Mérida, Mexico, found that after individually testing *Ae. aegypti* mosquitoes only 66 females out of 10,254 (<1%) were positive for DENV [29]. These findings outline a common issue with population-wide cross-sectional quantifications of ABV infection: the natural infection rate of an *Ae. aegypti* population may not be a function of where *Ae. aegypti* are, but rather where key human-mosquito contacts occur [34]. The possibility for early detection of virus circulation within a population may be key in preventing outbreaks, providing a cost-effective approach for triggering vector control. In a study conducted in Guerrero, Mexico, circulation of CHIKV was detected 10 days before any reported symptomatic human case, which allowed for early vector control actions and outbreak mitigation [7, 35].

The capacity of capturing a considerable and representative sample of mosquitoes is necessary for a comprehensive characterization of their natural infection. A myriad of adult *Ae. aegypti* sampling methods have been used for quantifying ABV natural infection rate. While passive traps (BG sentinel,

sticky ovitraps, Gravid *Aedes* traps, autocidal *Aedes* gravid ovitrap [36]) may allow for widespread coverage, they also require multiple days for capturing enough mosquitoes for virus testing and their sensitivity to vector and virus detection is unknown. Adult aspiration, while it is assumed to be more laborious and dependent on trained staff, provides an instantaneous measure of vector density and is considered a gold standard for adult *Ae. aegypti* collection [36, 37]. Applying sequential removal sampling using Prokopack aspirators [18, 37] the absolute density of *Ae. aegypti* was found to be up to five times bigger than previously estimated implementing the standard 10-minute collection period per household. As all studies quantifying ABV infection in *Ae. aegypti* have sampled a small fraction of the adult population and pooled collected mosquitoes to increase yield and detectability, there is a significant knowledge gap with regards to the magnitude of natural ABV infection rates within areas of active transmission.

There is a need for improving the evidence base of the epidemiological impact of vector control on ABV [38] . Estimates of ABV infection in *Ae. aegypti* infection could be calculated as measures of intervention impact, provided they are accurately quantified. In preparation for a clinical trial evaluating the epidemiological impact of targeted indoor residual spraying (TIRS) on ABVs [39], here we extended an observational study that used exhaustive Prokopack collections to quantify absolute *Ae. aegypti* density in houses with suspected active virus transmission [18], to quantify absolute ABV infection rate in individual *Ae. aegypti*. As a secondary analysis, we used this dataset to quantify the sensitivity of indoor adult *Ae. aegypti* collections using Prokopack aspirators in detecting ABV-infected mosquitoes, and quantified measures transmission potential.

Material and Methods

Study area and design

The study was conducted in Merida (population ~1 million), Yucatan, Mexico. Merida is endemic for dengue [3, 4, 40] and, as most of the Americas, was recently and sequentially invaded by CHIKV and ZIKV [14]. Arbovirus transmission is seasonal, peaking during the rainy season (July-November). Since 2011, Merida is home of a longitudinal cohort study called "Familias sin Dengue" (FSD, Families without dengue) that has characterized arbovirus infection and seroconversion rates and the entomological correlates of dengue infection [3, 4, 40]. Our study design originally involved selecting a total of 200 houses within FSD city blocks where recent (within 1 month) CHIKV, ZIKV or DENV occurred [18]. Surveillance for symptomatic cases occurred between June and December for two transmission seasons (2016-2017). Given the protocols for human subjects and household access, the team receive a list of houses without information of how many individuals were infected (or when onset of symptoms occurred) or the virus infecting them. Therefore, the entomological team only had a list of houses to visit, and they were blind to any information about arbovirus infection status or intensity in each house. Collections occurred in a period of ABV transmission in Merida, with DENV and CHIKV being reported to the city's passive surveillance system in 2015, and the introduction of ZIKV since 2016 onwards (Fig. S1). After obtaining informed consent from householders, exhaustive adult mosquito collections with Prokopack aspirators [22] were conducted using removal sampling, as described by Koyoc-Cardeña et al. [18]. Briefly, trained fieldworke[illegible]equentially entered each house and collected mosquitoes from each room (including the kitchen and bathroom). Removal sampling was conducted with a constant effort at predefined intervals of 10 min over the course of three hours or, if during two consecutive rounds no *Ae. aegypti* were captured.

Collected mosquitoes were transported alive to the Autonomous University of Yucatan entomology lab (UCBE-UADY) and immobilized at −20 °C for 10 min for sexing and taxonomical identification using standard keys. Additionally, blood-fed female *Ae. aegypti* were classified by the degree of blood digestion according to the Sella scale [41, 42], which was extended to include recent

feeding as a category (the presence of bright red blood was indicative of blood feeding within 24h of collection, and assigned a category '2' of Sella). Finally, male and female *Ae. aegypti* were individually dissected, their heads and bodies were separated and preserved in 1.5ml vials containing RNALater (Thermo Fisher Scientific, Waltham, MA, USA) with 1.5µl Tween® 20 (Sigma-Aldrich Co.) and stored at -20°C for future virus detection by molecular methods.

Ethics Statement

Protocols for this study were approved by Emory University's ethics committee under protocol ID: IRB00082848. The protocol was also approved by the Ethics and Research Committee from the O´Horan General Hospital from the state Ministry of Health, Register No. CEI-0-34-1-14. Written informed consent was obtained from the head of household prior to mosquito collection.

Detection of arboviral infections in *Ae. aegypti*

Initially, RNA was extracted from bodies (thorax, abdomen and extremities). Individual specimens were homogenized using a cordless motor tissue distributor (Kimble®) in a 1.5ml microcentrifuge tube with 150µl of PBS 1X, p.H 7.2 (GIBCO®) and centrifuged at 4°C for 10 minutes at 1,500g. Total RNA was extracted from 140µl of the mosquito's body disruption supernatant using QIAamp Viral RNA Mini Kit (QIAGEN®) following the manufacturer's recommendations. Finally, extracted RNA was eluted with 40µl of RNA-ase free water and preserved at -80°C. RNA extraction from heads was performed only from bodies that were positive for any of the targeted virus.

Detection of viral RNA was carried out by real-time RT-PCR using a probe-based detection method with a QuantiFast Probe RT-PCR Kit (QIAGEN®). RT-PCR reactions were performed in a Step One Plus Real-Time PCR System (Applied Biosystems®) following standard protocols. Reactions (samples) were considered positive when a sigmoidal curve was detected at a Ct value ≤38 cycles of amplification.

Table S1 shows the Primers and probes used to target CHIKV, ZIKV [43, 44] and DENV (personal communication from Davis Arbovirus Research & Training).

Positive samples for CHIKV and ZIKV were reconfirmed by end-point RT-PCR using a high-fidelity polymerase, SuperScript™ III One-Step RT-PCR System with Platinum™ *Taq* DNA polymerase (Thermo Fisher Scientific). Primers were specifically designed to target a 420bp fragment of the viral gene E1 of CHIKV (including the M13 universal sequence, underlined): Fwd (5' – TGTAAAACGACGGCCAGTAGACGTCTATGCTAATACACAACTG - 3') and Rev (5' – CAAGAAACAGCTATGACCTGAGAATTCCCTTCAACTTCTATCT - 3'); or a fragment of 662 bp of the viral gene NS1 of ZIKV (primers were kindly provided by MSc. Jesus Reyes and are available upon request). PCR positive amplicons were sequenced for molecular confirmation of virus presence. For DENV, sequencing was performed on the amplicons obtained from the qRT-PCR, corresponding to a fragment of 212 bp of the NS5 viral gene. Samples with evidence of ABV infection by qRT-PCR were sent to Macrogen corp® and sequenced by Sanger Method.

Sequence analysis

Single forward and reverse raw sequencing data were assessed based on quality score. Reads were compared to those from the GenBank database using NCBI BLASTN (https://blast.ncbi.nlm.nih.gov/Blast.cgi) at default parameters (Madden 2013). BLAST "hits" were used to assign reads to virus type, statistical significance was measured by the E-value and percentage or coverage. Reads that did not fulfill these conditions were considered potential chimeric sequences and discarded. Visualization of electropherograms, nucleotide sequences manipulation, alignment and analysis were performed using the software *Genious* Prime 2020.0.4 [45].

Data analysis

In the context of this study, absolute *Ae. aegypti* density per house (termed total catch) was calculated as the sum of adult females collected across all sampling rounds, whereas relative density was calculated as the number of females per unit time (e.g., 10 minutes). For analyses, houses were categorized based on their *Ae. aegypti* female total catch as high (≥ 10 collected) or low (<10 collected), as in Koyoc-Cardeña et al. [18]. Absolute natural infection rate was calculated as the total number of infected females divided by the total catch per house, whereas relative natural infection rate was calculated as the number of infected and collected *Ae. aegypti* within a given unit of collection time (e.g., 10-minutes). The sensitivity of the adult aspiration to the detection of infected *Ae. aegypti* mosquitos was estimated by plotting the cumulative relative natural infection rate as a function of the collection time (catch effort). Chi-squared tests were used to compare infection rates by house, based on their density category (low vs high). To quantify the relationship between female adult *Ae. aegypti* density (count variable) and ABV infection (binary variable: infected = 1, not infected = 0) at the house level, generalized linear mixed models with a binomial link function and a random intercept associated with each house ID were employed, as described in Vazquez-Prokopec et al. [20]. The same model was extended to include other predictor variables, such as the presence of a blood meal in the mosquito (binary) or the Sella engorgement score of females (categorical). Akaike information criterion (AIC) was used to identify the best model, among all models tested [46].

Two measures of ABV transmission potential were calculated using individual-level estimates of biting probability, infection, and vector density. The Entomological Inoculation Rate (EIR, expressed as the number of potentially infectious bites per person per day), routinely calculated for malaria [47], is considered a reliable measure of human exposure to infectious mosquitoes.

We calculated the EIR of ABVs at the household-level using the following equation: $IP = mas;$ where *m* is the ratio of *Ae. aegypti* females to the number of residents of each house, *a* is the number of bites per day (calculated as the ratio of *Ae. aegypti* females with Sella's score 2 by the total number of *Ae.*

aegypti females per house; Sella's score 2 indicates evidence of a bloodmeal within 24hs of capture) and *s* is the proportion of *Ae. aegypti* females found infected with any ABV.

Vectorial capacity (VC) is a common metric that estimates the number of infectious vectors generated from a population feeding on a single infected host per unit area/time [48]. Here, we estimated the daily VC of ABVs per house, as follows: $VC = \frac{ma^2p^n}{-Ln(p)}$, where *m* and *a* are equivalent as in EIR and *p* is the daily survival probability of female mosquitoes (set as p=0.7) and *n* the extrinsic incubation period (set as n=1/5 days).

We calculated both EIR and VC for the total catch as well as the first round and conducted paired t-test to evaluate the difference in their value between samples by house. A GLMM with a Gaussian link function and random effect at the house level was applied to evaluate the association between each metric (EIR, VC set as dependent variables) and the total catch of *Ae. aegypti* by house.

All analyses were performed within the R programing environment (https ://www.r-proje ct.org/) and GAMMs were run using the *lme4* package [34].

Results

Characteristics of ABV-infected *Ae. aegypti*

A total of 3,439 *Ae. aegypti* were collected in 179 houses, with 2,161 being females (62.8%). Of all collected females, 166 (7.7%) were positive for arbovirus infection (Table 1). The majority of infections were identified as positive for CHIKV (77.7%), followed by DENV (11.4%) and ZIKV (9.0%); coinfection with CHIKV and ZIKV was detected in three mosquitoes (1.8%) (Table 1). Of the total ABV-infected females, 38 (22.9%) had evidence of infection in their heads; 33 (86.8%) of them were positive for CHIKV, 1 (2.6%) for ZIKV, and 1 (2.6%) for DENV (Table 2). Additionally, 3 (7.9%) coinfections with CHIKV and ZIKV were detected in three heads, which correspond to coinfections also detected in their bodies (Table 2).

Out of the total number of female mosquitoes, 81.3% were blood feed, at different blood feeding status (Sella's score), with 26.0% of them being fed withing 24-h of collection (Sella's score 2). The majority of positive females were blood engorged at the different blood feeding status (86.1%), with 34.3% freshly feed (Sella 2; Table 3). The remaining 33.1% of infected females were either unfed (19.3% - Sella 1) or gravid (13.2% - Sella 7) (Table 3). A 7.2% (n=12) of the positive heads corresponded to positive bodies of female mosquitoes that were also classified with Sella score 2 (Table 3).

Natural ABV infection rate of female *Ae. aegypti*

At the house level and when using the total catch of *Ae. aegypti*, ABV infections were detected in 43 houses (25.4%) out of 169 houses infested with female mosquitoes. In those 43 houses, ABV infections were divided as follow: 37.2% for CHIKV, 27.9% for DENV, and 25.6% for ZIKV (Table 1). Additionally, co-occurrence of mosquitoes infected with any of the three viruses was detected in 3 houses (7.0%) and 3 specimens of *Ae. aegypti* mosquitos co-infected with CHIKV and ZIKV were found in a single house (2.3%) (Table 1). The median of infected mosquitoes per positive houses was 1 (interquartile range [IQR]= 4-1). The distribution of positive females per house varied by virus, and for CHIKV was highly skewed with a maximum of 25 CHIKV infected *Ae. aegypti* in one house (Fig. 1). The high overdispersion was further evidenced by the finding of 32.6% of houses contributing with 81.3% of the infected mosquitoes (Fig. 1).

A significantly higher proportion of houses were found infected by any ABV in the high-density group (42.9%) compared to the low-density group (13.1%) ($X^2_{(df=1)}$ = 17.6, P <0.001). When mosquito density was high, a larger proportion of houses had mosquitoes infected with CHIKV (18.6%) compared to DENV (12.9%) or ZIKV (8.6%); a 4.3% co-occurrence of infected mosquitoes with either virus was observed in high density houses. Comparatively, there was a similar proportion of houses with positive mosquitoes for each virus when mosquito density was low (Fig. 2A). When analyzing mosquitoes with

positive heads, only 3.0% were found in low-density houses while positive mosquito heads were found in 18.6% of high-density houses (Fig. 2B). The probability of finding infected *Ae. aegypti* was significantly associated with absolute density indoors (binomial GLMM (Odds Ratio [95% CI]): 1.0 [1.0 – 1.1]), with houses having more than 40 *Ae. aegypti* females having a probability infection above 60% (Fig. 3). When only considering infected female heads, no association with absolute density was found (1.0 [0.9 - 1.1]). Sella score did not have any significant association with infection for all adults or infected heads (Table S2).

Fig. 4 shows the sensitivity of Prokopack collections to the detection of ABV infected *Ae. aegypti* females. Performing a single 10-min Prokopack collection indoors led to a low (16.3%) sensitivity of detecting an ABV infected house (Fig. 4A) or infected female (23.4%) (Fig. 4B). The low sensitivity translated to each individual virus, both for houses (15.0% for CHIKV, 5.3% for DENV and, 25.0% for ZIKV) and individual mosquitoes (25.9% for CHIKV, DENV 5.3% and ZIKV 23.8%) (Fig. 4). As collection time increased, the sensitivity of detection increased both for houses and mosquitoes, reaching an asymptote at ~120 min for any viral infection (Fig. 4). Aggregating data from the first two sampling rounds (i.e. equivalent to performing a 20-min collection) led to an increase in household infection sensitivity (+16.3% for any adults, +15.0% for CHIKV, +26.3% for DENV and +15.0% for ZIKV; Fig. 4A) and individual mosquito sensitivity (+17.5% for any adults, +16.8% for CHIKV, +26.3% for DENV and +14.3% for ZIKV; Fig. 4B).

Estimates of ABV transmission potential

The ratio of *Ae. aegypti* females to humans (*m*) increased significantly between the sampled mosquito density and the total catch (paired t-test = 6.4312, df = 199, $p < 0.001$; Fig. 5A, Table S3). At densities higher than 4 *Ae. aegypti* females, a GLMM predicted *m* would surpass one; the maximum observed *m* was 30 mosquitoes per person (Fig. 5B). When averaged across all infested houses, mean

EIR ranged between 0.04 and 0.06 infective bites per person per day, with estimates for total catch and sample being not statistically significant (paired t-test = -1.2988, df = 103, p = 0.1969) (Fig. 5C, Table S3). When only houses with infected *Ae. aegypti* were considered, mean EIR for the total catch increased to 0.28 infectious bites per person per day (Standard Deviation = 0.36; range =0.01-1.5). Increasing total catch indoors lead to slight predicted variation in EIR (Fig. 5D) likely due to the low infection rate (parameter *s*).

ABV transmission potential, measured as mean VC, was significantly higher for the total catch than the sample (t = -2.6487, df = 103, p-value = 0.009) (Fig. 5E). When scaled by total catch indoors, VC showed a significant increase from 1.0 below 20 *Ae. aegypti* females per house to 3.0 at density of 70 mosquitoes per house; the maximum VC estimate registered was 5.9 (Fig. 5F, Table S3).

Large variability in feeding frequency (Fig. S2) influenced estimates of VC and EIR, for instance a house with a total catch of 60 had only 2 females *Ae. aegypti* with Sella's score 2, leading to a low estimate of parameter *a*.

Nucleotide sequence analysis

Sanger sequencing confirmed with high fidelity the presence the three ABVs targeted by RT-PCR (Table S4). High-quality reads matched perfectly or nearly perfectly (BLASTn search hit >90% identity) to CHIKV, DENV and ZIKV genomes published in NCBI GenBank. Consensus sequences were assembled for most samples sequenced for CHIK and ZIKV and will be used for future phylogenetic analysis. For DENV, ten single strand sequences confirmed DENV type 4 serotype as the circulating serotype (Table S4). The virus identity of all positive heads matched the identity of the virus for the corresponding positive bodies (Table S4).

Discussion

CHIKV, DENV and ZIKV transmission risk appears to be correlated with the vector density and the number of infected mosquitoes at a coarse scale (entire cities, sub-national units) [25, 26], but such association between entomological indices and *ABV* incidence is generally inconsistent at the local level [26, 31, 49]. Our findings show that sampling bias in the quantification of vector density and in virus detection sensitivity as well as strong overdispersion in the distribution of infected mosquitoes may be important contributors to such inconsistency. We found that the sensitivity of routine Prokopack collections (10 min per house), considered a gold standard for indoor adult *Ae. aegypti* collections, in detecting houses with infected *Ae. aegypti* mosquitoes was below 25%. Furthermore, when infection was quantified in the total catch, approximately 80% of all infected mosquitoes were collected from ~30% of infested houses. Both findings are relevant for the design of sampling schemes aimed at entomo-virological surveillance of *Ae. aegypti*, as it is evident that detecting infected mosquitoes will be a function of the collection method used, the sampling effort, the local abundance of *Ae. aegypti* and the intensity of arbovirus circulation.

In our previous study, we quantified that houses may harbor up to five times more adult *Ae. aegypti* than estimated during routine Prokopack collections [18]. These data show that the low apparent density of *Ae. aegypti* indoors [50] may also be a function of the sensitivity of the collection method. The ability of *Ae. aegypti* to feed frequently (~1.5 days) and of distributing bites on some individuals more than others (aka., heterogeneous biting) [51-53] are considered the mechanisms compensating for the low *Ae. aegypti* density and human-mosquito rates [15]. Here we show that including the total *Ae. aegypti* population indoors (in our study, by factor of 5x) significantly elevates human - mosquito contacts and can have profound effects on estimates of natural infection and ABV transmission risk. While we did not directly measure heterogeneous biting (which requires DNA profiling in mosquitoes and humans), we evidenced its powerful epidemiological effect in the strong overdispersion of infection in collected mosquitoes. Seven houses harbored more than 10 CHIVK

infected females each, with one having up to 25 infected females. Aggregation of bites on one or a few infected individuals is the most likely explanation for such remarkable aggregation of infection. As transmission of ABVs is shaped by the daily mobility patterns of humans [19, 54], any residents or visitors to such 'key locations' may experience a disproportionately high risk of infection. Evaluating the impact of observed total density of *Ae. aegypti* per house (which may well reach 100 females per house, [18]) on ABV transmission dynamics may help understand both the stability of virus transmission chains and the impact of novel interventions focused on the indoor adult population.

Several innovative strategies are being evaluated for their epidemiological impact on ABVs. Targeted Indoor Residual Spraying (TIRS), capitalizes on indoor resting behavior of *Ae. aegypti* (which primarily is found resting below 1.5 m and in dark surfaces) to deliver long-lasting residual insecticides that can significantly reduce vector density and dengue transmission [55, 56]. *Wolbachia* population replacement or suppression approaches rely on the release of adults and capitalize their mating strategy to either render the population incompetent to transmission or reduce adult female density, respectively [57]. Spatial repellents are volatilized pyrethroids that disrupt mosquito behavior and reduce human-mosquito contacts indoors, without apparent impact on population density [58]. All such approaches are dependent on an accurate characterization of the population density of the vector (for instance, release rates need accurate density estimates, repellency may not be effective at high vector numbers, residual effect may increase evolution of resistance at high densities) and careful monitoring of their future implementation will require quantifying their effect on ABV infection.

A randomized controlled clinical trial will evaluate the epidemiological impact of TIRS in Merida [39], with ABV infection in *Ae. aegypti* being quantified as a secondary endpoint. Our findings suggest that entomological collections with Prokopacks indoors should be conducted for more than 10 minutes per house. Increasing the collection effort will increase the probability of detecting ABV infected *Ae. aegypti*. In the context of the TIRS trial, obtaining accurate measures of ABV infection in *Ae. aegypti* will

lead to better estimates of the measured impact of the intervention, as it will allow quantifying what percent reduction in cases will be associated with a reduction in ABV infection in *Ae. aegypti* females. As other trials are implemented in the future, the consideration of the impact of an intervention on ABV infection in *Ae. aegypti* can be used to communicate vector control personnel the expected entomological effect of their actions.

Assessments of arbovirus infection in mosquitoes are commonly expressed as the prevalence of infections in pools of 15 - 30 individuals [26]. While MIR or MLR are commonly calculated, these indexes are prone bias particularly if infection aggregates within a household [26, 31, 32]. As far as we know, there is no study in which ABV infection has been explored in individual field collected mosquitoes. The only report we found, also conducted in Mérida, used RNA extracted from individual females *Ae. aegypti* and tested for DENV infection by RT-PCR in pools of 10 extractions, with mosquitoes from positive pools further tested individually [29]. Such study, which occurred in periods of high and low DENV transmission, found natural infection rates of <1%. Our study found high prevalence of infection by CHIKV in *Ae. aegypti* mosquitoes in a period when the most reported infection in Merida was ZIKV (Sup. Fig 1). Considering that most ABV infections go undetected to the public health system, either as asymptomatic or subclinical infections or for mild illness, may help explain the mismatch between high CHIKV infection in mosquitoes and the focus on ZIKV testing during this period of virus introduction into Merida [14]. There are reports of early detection of CHIKV and ZIKV infection in *Ae. aegypti* from other states of Mexico prior to the detection of symptomatic cases [59, 60], which supports the known assumption that passive surveillance may fail to detect virus circulation in periods of low transmission.

We also found houses infested with mosquitoes positive for different viruses, suggesting the co-circulation of more than one virus within the area and even within the same house. CHIKV and ZIKV-positive mosquitoes were found in two houses, while CHIKV and DENV-positive specimens were

detected in one house. Additionally, coinfections of CHIKV and ZIKV was detected in three specimens within the same house. This data aligns with other studies that also reported the cohabitation of mosquitoes infected with different viruses within the same area or houses and the coinfection of two (or more) different viruses in individual mosquito. Cases of humans co-infected with multiple viruses have been reported in the Americas [61] and other regions [62-64]. Coinfections with all 3 arboviruses—CHIKV, DENV, and ZIKV—have also been reported [65, 66]. *Aedes aegypti* infected with more than one virus has also been detected, for example mosquitoes coinfected with ZIKV and DENV were detected in Manaus, showing that ZIKV is preferentially transmitted over DENV when in coinfection [67]. Coinfection and transmission capacity of DENV/ CHIKV was also demonstrated thought experimental infection of *Ae. aegypti* [68]. Notwithstanding, the epidemiological impact of multiple infections is unknown.

In order to accurately confirm the detected ABV infection, we sequenced every PCR-positive sample. Our sequence reads positively confirmed the PCR results. In the case of DENV, we were able to typify the virial serotype as DENV-4. This result line up with previous results obtained from different work in the area where all four DENV serotypes were found circulating in Merida being DENV-4 the predominated in the most years with DENV-1 and DENV-3 [14, 69].

By individually testing mosquitoes, and individually testing body parts (head or abdomen) we unveiled important details about the process of infection and human-mosquito contacts in *Ae. aegypti*. The majority of recently blood fed females (Sella score 2) were positive for CHIKV (34.3%). We also detected 32 (19.3%) unfed (Sella score 1) infected females, which this could be interpreted as females that had blood fed and digested the blood ready for another gonotrophic cycle. Generally, a mosquito is considered to be infective when especially the head is positive, indicating the infection of the salivary glands by the virus and ready to be transmitted through the next blood meal. We found 38 female specimens with positive head, 86.8% of those were CHIKV-positive, and 5 specimens were head positive and unfed status, presumably infectives. Gravid females (Sella's score 7) with positive heads were also

detected (7 specimens). We used such information to innovatively calculate indices of transmission potential or risk (VC or EIR) not commonly estimated for ABVs. We found that transmission potential (VC) was sensitive to the total density of mosquitoes collected, whereas transmission risk (EIR) was sensitive to the detection of infected mosquitoes. Our analyses indicate that when *Ae. aegypti* total density is calculated, a significant association with two measures of ABV transmission exists. Such findings highlight the relevance of accurate estimates of vector density and infection rates and highlight the potential for metrics such as EIR or VC to be used as endpoints for the evaluation of the impact of vector control on ABVs.

Acknowledgements

We thank the residents of Merida, Yucatan, for kindly allowing us to conduct this important research.

Author contributions

Conceptualization: GVP, PMS, GAT, ODK, NPR

Formal Analysis: ODK, GVP, GAT, DCE

Methodology: NPR, EKC, ACM, ACR, PGP, HPG, MWD

Project Administration: PMS, GVP

Manuscript preparation: ODK, GVP, PMS

Review & Editing All authors

Funding Statement

Research funding was provided by an Interagency Agreement between USAID and the US Centers for Disease Control and Prevention (CDC: OADS BAA 2016-N-17844; PI, Vazquez-Prokopec G.M.), by the Canadian Institutes of Health Research (CIHR) and IDRC (Preventing Zika disease with novel vector control approaches, Project 108412) by Fondo Mixto CONACyT (Mexico)-Gobierno del Estado de Yucatan (Project YUC-2017-03-01-556), the National Institutes of Health (NIH/NIAID: U01AI148069) and Emory University via the MP3 initiative (Vazquez-Prokopec, PI). The opinions expressed herein are those of the author(s) and do not necessarily reflect the views of Emory University or the U.S. Agency for International Development. The findings and conclusions in this paper are those of the authors and do not necessarily represent the official position of the Centers for Disease Control and Prevention.

References

1. Pistone T, Ezzedine K, Schuffenecker I, Receveur M-C, Malvy D. An imported case of Chikungunya fever from Madagascar: use of the sentinel traveller for detecting emerging arboviral infections in tropical and European countries. Travel medicine and infectious disease. 2009;7(1):52-4.

2. Dash A, Bhatia R, Sunyoto T, Mourya D. Emerging and re-emerging arboviral diseases in Southeast Asia. Journal of vector borne diseases. 2013;50(2):77.

3. Pavía-Ruz N, Contreras-Capetillo S, Valadéz-González N, Villegas-Chim J, Carcaño-Castillo R, Valencia-Pacheco G, et al. An Integrated Intervention Model for the Prevention of Zika and Other Aedes-Borne Diseases in Women and their Families in Mexico. Current Topics in Zika: IntechOpen; 2017.

4. Rojas DP, Barrera-Fuentes GA, Pavia-Ruz N, Salgado-Rodriguez M, Che-Mendoza A, Manrique-Saide P, et al. Epidemiology of dengue and other arboviruses in a cohort of school children and their families in Yucatan, Mexico: Baseline and first year follow-up. PLoS neglected tropical diseases. 2018;12(11).

5. Aragão CF, Pinheiro VCS, Nunes Neto JP, Silva EVPd, Pereira GJG, Nascimento BLSd, et al. Natural Infection of Aedes aegypti by Chikungunya and Dengue type 2 Virus in a Transition Area of North-Northeast Brazil. Viruses. 2019;11(12):1126.

6. Zanotto PMdA, Leite LCdC. The challenges imposed by Dengue, Zika, and Chikungunya to Brazil. Frontiers in immunology. 2018;9:1964.

7. Dzul-Manzanilla F, Martinez NE, Cruz-Nolasco M, Gutierrez-Castro C, Lopez-Damian L, Ibarra-Lopez J, et al. Evidence of vertical transmission and co-circulation of chikungunya and dengue viruses in field populations of Aedes aegypti (L.) from Guerrero, Mexico. Trans R Soc Trop Med Hyg. 2016;110(2):141-4. Epub 2015/12/30. doi: 10.1093/trstmh/trv106. PubMed PMID: 26711697.

8. Dos Reis IC, Gibson G, Ayllón T, de Medeiros Tavares A, de Araújo JMG, da Silva Monteiro E, et al. Entomo-virological surveillance strategy for dengue, Zika and chikungunya arboviruses in field-caught Aedes mosquitoes in an endemic urban area of the Northeast of Brazil. Acta tropica. 2019;197:105061.

9. Beltrán-Silva S, Chacón-Hernández S, Moreno-Palacios E, Pereyra-Molina J. Clinical and differential diagnosis: Dengue, chikungunya and Zika. Revista Médica del Hospital General de México. 2018;81(3):146-53.

10. Shapiro-Mendoza CK, Rice ME, Galang RR, Fulton AC, VanMaldeghem K, Prado MV, et al. Pregnancy outcomes after maternal Zika virus infection during pregnancy—US Territories, January 1, 2016–April 25, 2017. MMWR Morbidity and mortality weekly report. 2017;66(23):615.

11. Hawman DW, Stoermer KA, Montgomery SA, Pal P, Oko L, Diamond MS, et al. Chronic joint disease caused by persistent Chikungunya virus infection is controlled by the adaptive immune response. Journal of virology. 2013;87(24):13878-88.

12. Gu W, Unnasch TR, Katholi CR, Lampman R, Novak RJ. Fundamental issues in mosquito surveillance for arboviral transmission. Transactions of the Royal Society of Tropical Medicine and Hygiene. 2008;102(8):817-22.

13. Achee NL, Grieco JP, Vatandoost H, Seixas G, Pinto J, Ching-Ng L, et al. Alternative strategies for mosquito-borne arbovirus control. PLoS neglected tropical diseases. 2019;13(1).

14. Bisanzio D, Dzul-Manzanilla F, Gomez-Dantés H, Pavia-Ruz N, Hladish TJ, Lenhart A, et al. Spatio-temporal coherence of dengue, chikungunya and Zika outbreaks in Merida, Mexico. PLoS neglected tropical diseases. 2018;12(3):e0006298.

15. Scott TW, Morrison AC. Aedes aegypti density and the risk of dengue-virus. Ecological aspects for application of genetically modified mosquitoes. 2003;2:187.

16. Bowman LR, Runge-Ranzinger S, McCall P. Assessing the relationship between vector indices and dengue transmission: a systematic review of the evidence. PLoS neglected tropical diseases. 2014;8(5).

17. Cromwell EA, Stoddard ST, Barker CM, Van Rie A, Messer WB, Meshnick SR, et al. The relationship between entomological indicators of Aedes aegypti abundance and dengue virus infection. PLoS neglected tropical diseases. 2017;11(3).

18. Koyoc-Cardeña E, Medina-Barreiro A, Cohuo-Rodríguez A, Pavía-Ruz N, Lenhart A, Ayora-Talavera G, et al. Estimating absolute indoor density of Aedes aegypti using removal sampling. Parasites & vectors. 2019;12(1):250.

19. Stoddard ST, Forshey BM, Morrison AC, Paz-Soldan VA, Vazquez-Prokopec GM, Astete H, et al. House-to-house human movement drives dengue virus transmission. Proceedings of the National Academy of Sciences. 2013;110(3):994-9.

20. Vazquez-Prokopec GM, Medina-Barreiro A, Che-Mendoza A, Dzul-Manzanilla F, Correa-Morales F, Guillermo-May G, et al. Deltamethrin resistance in Aedes aegypti results in treatment failure in Merida, Mexico. PLoS neglected tropical diseases. 2017;11(6):e0005656.

21. Reiner RC, Stoddard ST, Forshey BM, King AA, Ellis AM, Lloyd AL, et al. Time-varying, serotype-specific force of infection of dengue virus. Proceedings of the National Academy of Sciences. 2014;111(26):E2694-E702.

22. Overgaard HJ, Pientong C, Thaewnongiew K, Bangs MJ, Ekalaksananan T, Aromseree S, et al. Assessing dengue transmission risk and a vector control intervention using entomological and immunological indices in Thailand: study protocol for a cluster-randomized controlled trial. Trials. 2018;19(1):122.

23. Barrera R, Amador M, Acevedo V, Beltran M, Munoz J. A comparison of mosquito densities, weather and infection rates of Aedes aegypti during the first epidemics of Chikungunya (2014) and Zika (2016) in areas with and without vector control in Puerto Rico. Medical and veterinary entomology. 2019;33(1):68-77.

24. Kuno G. Factors influencing the transmission of dengue viruses. Dengue and dengue hemorrhagic fever. 1997;1:23-39.

25. Scott TW, Morrison AC. Vector dynamics and transmission of dengue virus: implications for dengue surveillance and prevention strategies. Dengue virus: Springer; 2010. p. 115-28.

26. Bustamante DM, Lord CC. Sources of error in the estimation of mosquito infection rates used to assess risk of arbovirus transmission. The American journal of tropical medicine and hygiene. 2010;82(6):1172-84.

27. Gordon SW, Tammariello RF, Linthicum KJ, Dohm DJ, Digoutte J, Calvo-Wilson M. Arbovirus isolations from mosquitoes collected during 1988 in the Senegal River basin. The American journal of tropical medicine and hygiene. 1992;47(6):742-8.

28. Faye O, Faye O, Diallo D, Diallo M, Weidmann M. Quantitative real-time PCR detection of Zika virus and evaluation with field-caught mosquitoes. Virology journal. 2013;10(1):311.

29. Loroño-Pino MA, García-Rejón JE, Machain-Williams C, Gomez-Carro S, Nuñez-Ayala G, del Rosario Nájera-Vázquez M, et al. Towards a Casa Segura: a consumer product study of the effect of insecticide-treated curtains on Aedes aegypti and dengue virus infections in the home. The American journal of tropical medicine and hygiene. 2013;89(2):385-97.

30. Coffey LL, Page BL, Greninger AL, Herring BL, Russell RC, Doggett SL, et al. Enhanced arbovirus surveillance with deep sequencing: Identification of novel rhabdoviruses and bunyaviruses in Australian mosquitoes. Virology. 2014;448:146-58.

31. CHIAN[illegible]-l, Reeves WC. Statistical estimation of virus infection rates in mosquito vector populations. American Journal of Hygiene. 1962;75(3).

32. Gu W, Lampman R, Novak RJ. Problems in estimating mosquito infection rates using minimum infection rate. Journal of medical entomology. 2003;40(5):595-6.

33. Gu W, Lampman R, Novak R. Assessment of arbovirus vector infection rates using variable size pooling. Medical and veterinary entomology. 2004;18(2):200-4.

34. Anders KL, Le Hong Nga NTV, Thuy TVN, Tam CT, Tai LTH, Truong NT, et al. Households as foci for dengue transmission in highly urban Vietnam. PLoS neglected tropical diseases. 2015;9(2).

35. Dzul-Manzanilla F, Martínez NE, Cruz-Nolasco M, Gutiérrez-Castro C, López-Damián L, Ibarra-López J, et al. Arbovirus surveillance and first report of chikungunya virus in wild populations of Aedes aegypti from Guerrero, Mexico. Journal of the American Mosquito Control Association. 2015;31(3):275-7.

36. Silver JB. Mosquito ecology: field sampling methods: springer science & business media; 2007.

37. Vazquez-Prokopec GM, Galvin WA, Kelly R, Kitron U. A new, cost-effective, battery-powered aspirator for adult mosquito collections. Journal of medical entomology. 2009;46(6):1256-9.

38. Reiner Jr RC, Achee N, Barrera R, Burkot TR, Chadee DD, Devine GJ, et al. Quantifying the epidemiological impact of vector control on dengue. PLoS neglected tropical diseases. 2016;10(5):e0004588.

39. Clinical Trials. NIH - U.S. National Library of Medicine. Impact of Indoor Residual Spraying on Aedes-borne Diseases 2020 [cited 2020 10 July]. Available from: https://www.clinicaltrials.gov/ct2/show/NCT04343521.

40. Pavia-Ruz N, Barrera-Fuentes GA, Villanueva-Jorge S, Che-Mendoza A, Campuzano-Rincón JC, Manrique-Saide P, et al. Dengue seroprevalence in a cohort of schoolchildren and their siblings in Yucatan, Mexico (2015-2016). PLoS neglected tropical diseases. 2018;12(11).

41. Detinova TS, Bertram D, Organization WH. Age-grouping methods in Diptera of medical importance, with special reference to some vectors of malaria: World Health Organization; 1962.

42. Santos CS, Pie MR, da Rocha TC, Navarro-Silva MA. Molecular identification of blood meals in mosquitoes (Diptera, Culicidae) in urban and forested habitats in southern Brazil. PloS one. 2019;14(2).

43. Lanciotti RS, Kosoy OL, Laven JJ, Panella AJ, Velez JO, Lambert AJ, et al. Chikungunya virus in US travelers returning from India, 2006. Emerging infectious diseases. 2007;13(5):764.

44. Lanciotti RS, Kosoy OL, Laven JJ, Velez JO, Lambert AJ, Johnson AJ, et al. Genetic and serologic properties of Zika virus associated with an epidemic, Yap State, Micronesia, 2007. Emerging infectious diseases. 2008;14(8):1232.

45. Kearse M, Moir R, Wilson A, Stones-Havas S, Cheung M, Sturrock S, et al. Geneious Basic: an integrated and extendable desktop software platform for the organization and analysis of sequence data. Bioinformatics. 2012;28(12):1647-9.

46. Burnham KP, Anderson DR. A practical information-theoretic approach. Model selection and multimodel inference, 2nd ed Springer, New York. 2002.

47. Smith D, Dushoff J, Snow R, Hay S. The entomological inoculation rate and Plasmodium falciparum infection in African children. Nature. 2005;438(7067):492-5.

48. McMillan JR, Blakney RA, Mead DG, Koval WT, Coker SM, Waller LA, et al. Linking the vectorial capacity of multiple vectors to observed patterns of West Nile virus transmission. Journal of Applied Ecology. 2019;56(4):956-65.

49. Gu W, Novak RJ. Detection probability of arbovirus infection in mosquito populations. The American journal of tropical medicine and hygiene. 2004;71(5):636-8.

50. Scott TW, Morrison AC, Lorenz LH, Clark GG, Strickman D, Kittayapong P, et al. Longitudinal studies of Aedes aegypti (Diptera: Culicidae) in Thailand and Puerto Rico: population dynamics. Journal of medical entomology. 2000;37(1):77-88.

51. De Benedictis J, Chow-Shaffer E, Costero A, Clark GG, Edman JD, Scott TW. Identification of the people from whom engorged Aedes aegypti took blood meals in Florida, Puerto Rico, using polymerase chain reaction-based DNA profiling. The American journal of tropical medicine and hygiene. 2003;68(4):437-46.

52. Harrington LC, Fleisher A, Ruiz-Moreno D, Vermeylen F, Wa CV, Poulson RL, et al. Heterogeneous feeding patterns of the dengue vector, Aedes aegypti, on individual human hosts in rural Thailand. PLoS Negl Trop Dis. 2014;8(8):e3048.

53. Liebman KA, Stoddard ST, Reiner Jr RC, Perkins TA, Astete H, Sihuincha M, et al. Determinants of heterogeneous blood feeding patterns by Aedes aegypti in Iquitos, Peru. PLoS Negl Trop Dis. 2014;8(2):e2702.

54. Schaber KL, Paz-Soldan VA, Morrison AC, Elson WH, Rothman AL, Mores CN, et al. Dengue illness impacts daily human mobility patterns in Iquitos, Peru. PLoS neglected tropical diseases. 2019;13(9):e0007756.

55. Vazquez-Prokopec GM, Montgomery BL, Horne P, Clennon JA, Ritchie SA. Combining contact tracing with targeted indoor residual spraying significantly reduces dengue transmission. Science advances. 2017;3(2):e1602024.

56. Hladish TJ, Pearson CA, Toh KB, Rojas DP, Manrique-Saide P, Vazquez-Prokopec GM, et al. Designing effective control of dengue with combined interventions. Proceedings of the National Academy of Sciences. 2020;117(6):3319-25.

57. Flores HA, O'Neill SL. Controlling vector-borne diseases by releasing modified mosquitoes. Nature Reviews Microbiology. 2018;16(8):508-18.

58. Achee NL, Bangs MJ, Farlow R, Killeen GF, Lindsay S, Logan JG, et al. Spatial repellents: from discovery and development to evidence-based validation. Malaria Journal. 2012;11(1):164.

59. Dzul-Manzanilla F, Martínez NE, Cruz-Nolasco M, Gutiérrez-Castro C, López-Damián L, Ibarra-López J, et al. Evidence of vertical transmission and co-circulation of chikungunya and dengue viruses in field populations of Aedes aegypti (L.) from Guerrero, Mexico. Transactions of the Royal Society of Tropical Medicine and Hygiene. 2016;110(2):141-4.

60. Díaz-Quiñonez JA, López-Martínez I, Torres-Longoria B, Vázquez-Pichardo M, Cruz-Ramírez E, Ramírez-González JE, et al. Evidence of the presence of the Zika virus in Mexico since early 2015. Virus Genes. 2016;52(6):855-7.

61. Zambrano H, Waggoner JJ, Almeida C, Rivera L, Benjamin JQ, Pinsky BA. Zika virus and chikungunya virus coinfections: a series of three cases from a single center in Ecuador. The American journal of tropical medicine and hygiene. 2016;95(4):894-6.

62. Dupont-Rouzeyrol M, O'Connor O, Calvez E, Daures M, John M, Grangeon J-P, et al. Co-infection with Zika and dengue viruses in 2 patients, New Caledonia, 2014. Emerging infectious diseases. 2015;21(2):381.

63. Iovine NM, Lednicky J, Cherabuddi K, Crooke H, White SK, Loeb JC, et al. Coinfection with Zika and dengue-2 viruses in a traveler returning from Haiti, 2016: clinical presentation and genetic analysis. Clinical Infectious Diseases. 2016:ciw667.

64. Furuya-Kanamori L, Liang S, Milinovich G, Magalhaes RJS, Clements AC, Hu W, et al. Co-distribution and co-infection of chikungunya and dengue viruses. BMC infectious diseases. 2016;16(1):84.

65. Villamil-Gómez WE, Rodríguez-Morales AJ, Uribe-García AM, González-Arismendy E, Castellanos JE, Calvo EP, et al. Zika, dengue, and chikungunya co-infection in a pregnant woman from Colombia. International Journal of Infectious Diseases. 2016;51:135-8.

66. Waggoner JJ, Gresh L, Vargas MJ, Ballesteros G, Tellez Y, Soda KJ, et al. Viremia and clinical presentation in Nicaraguan patients infected with Zika virus, chikungunya virus, and dengue virus. Clinical Infectious Diseases. 2016:ciw589.

67. Chaves BA, Orfano AS, Nogueira PM, Rodrigues NB, Campolina TB, Nacif-Pimenta R, et al. Coinfection with Zika Virus (ZIKV) and Dengue Virus Results in Preferential ZIKV Transmission by Vector Bite to Vertebrate Host. The Journal of Infectious Diseases. 2018;218(4):563-71. doi: 10.1093/infdis/jiy196.

68. Nuckols J, Huang Y-J, Higgs S, Miller A, Pyles R, Spratt Hm, et al. Evaluation of simultaneous transmission of chikungunya virus and dengue virus type 2 in infected Aedes aegypti and Aedes albopictus (Diptera: Culicidae). Journal of medical entomology. 2015;52(3):447-51.

69. Pavía-Ruz N, Barrera-Fuentes GA, Villanueva-Jorge S, Che-Mendoza A, Campuzano-Rincón JC, Manrique-Saide P, et al. Dengue seroprevalence in a cohort of schoolchildren and their siblings in Yucatan, Mexico (2015-2016). PLoS neglected tropical diseases. 2018;12(11):e0006748.

70. Méndez N, Baeza-Herrera L, Ojeda-Baranda R, Huchim-Lara O, Gómez-Carro S. Perfil clinicoepidemiológico de la infección por Chikungunya en casos hospitalarios atendidos en 2015 en Mérida, México. Revista Panamericana de Salud Pública. 2017;41:e91.

Table 1. Descriptive measures and Infection rates in indoor resting *Ae. aegypti* mosquitoes from Yucatan, Mexico.

# of houses screened for *Ae. aegypti*	200
# of infested houses with *Ae. aegypti*	179 (89.5%)
# of infested houses with *Ae. aegypti* females	169 (94.4%)
Total # of *Ae. aegypti*	3,439
# of *Ae. aegypti* females	2,161 (62.8%)
# of *Ae. aegypti* males	1,278 (37.2%)
Sex ratio F:M	1.7:1
# of positive *Ae. aegypti* females for any virus	166 (7.7%)
# of positive *Ae. aegypti* females CH[illegible]KV	129 (77.7%)
# of positive *Ae. aegypti* females DENV	19 (11.4%)
# of positive *Ae. aegypti* females ZIKV	15 (9.0%)
# of positive *Ae. aegypti* females with coinfection CHIKV - ZIKV	3 (1.8%)
# of houses with positive *A. aegypti* females (+) for any virus	43 (25.4%)
# of houses (+) CHIKV	16 (37.2%)
# of houses (+) DENV	12 (27.9%)
# of houses (+) ZIKV	11 (25.6%)
# of houses (+) CHIV + ZIKV	1 (2.3%)
# of houses (+) CHIKV + DENV	2 (4.6%)
# of houses (+) with mosquito coinfection (CHIKV/ZIKV)	1 (2.3%)

Table 2 - Number of anatomical structures of *Ae. aegypti* mosquitoes infected with either virus collected in Yucatan, Mexico. Percentages indicate the fraction of infection with each virus for each anatomical structure.

Structure	DENV	CHIKV	ZIKV	CHIKV/ZIKV coinfection
**Head**	1 (2.6%)	33 (86.8%)	1 (2.6%)	3 (7.9%)
**Body**	18 (13.7%)	96 (73.3%)	14 (10.7%)	3 (2.3%)

Table 3 – Distribution of virus infection among Sella scores, and their relationship with positive heads from *Ae. aegypti* collected from Yucatan, Mexico**.**

Sella score	CHIKV	DENV	ZIKV	CHIKV/ZIKV	Total	Heads +
0	1 (0.6%)	0 (0.00%)	0 (0.00%)	0 (0.00%)	1 (0.60%)	1 (0.60%)
1	27 (16.3%)	2 (1.2%)	3 (1.8%)	0 (0.0%)	32 (19.3%)	5 (3.0%)
2	47 (28.3%)	6 (3.6%)	4 (2.4%)	0 (0.0%)	**57 (34.3%)**	12 (7.2%)
3	8 (4.8%)	5 (3.0%)	1 (0.6%)	0 (0.0%)	14 (8.4%)	3 (1.8%)
4	10 (6.0%)	0 (0.0%)	2 (1.2%)	2 (1.2%)	14 (8.4%)	4 (2.4%)
5	8 (4.8%)	2 (1.2%)	0 (0.0%)	0 (0.0%)	10 (6.0%)	1 (0.6%)
6	14 (8.4%)	2 (1.2%)	0 (0.0%)	0 (0.0%)	16 (9.6%)	5 (3.0%)
7	14 (8.4%)	2 (1.2%)	5 (3.0%)	1 (0.6%)	22 (13.2%)	7 (4.2%)
Total	129 (77.7%)	19 (11.4%)	15 (9.0%)	3 (1.8%)	166 (100%)	38 (23.0%)

Fig. 1 – Distribution of the number of female *Ae. aegypti* positive for CHIKV, DENV and ZIKV per house with positive mosquitoes from Yucatan, Mexico.

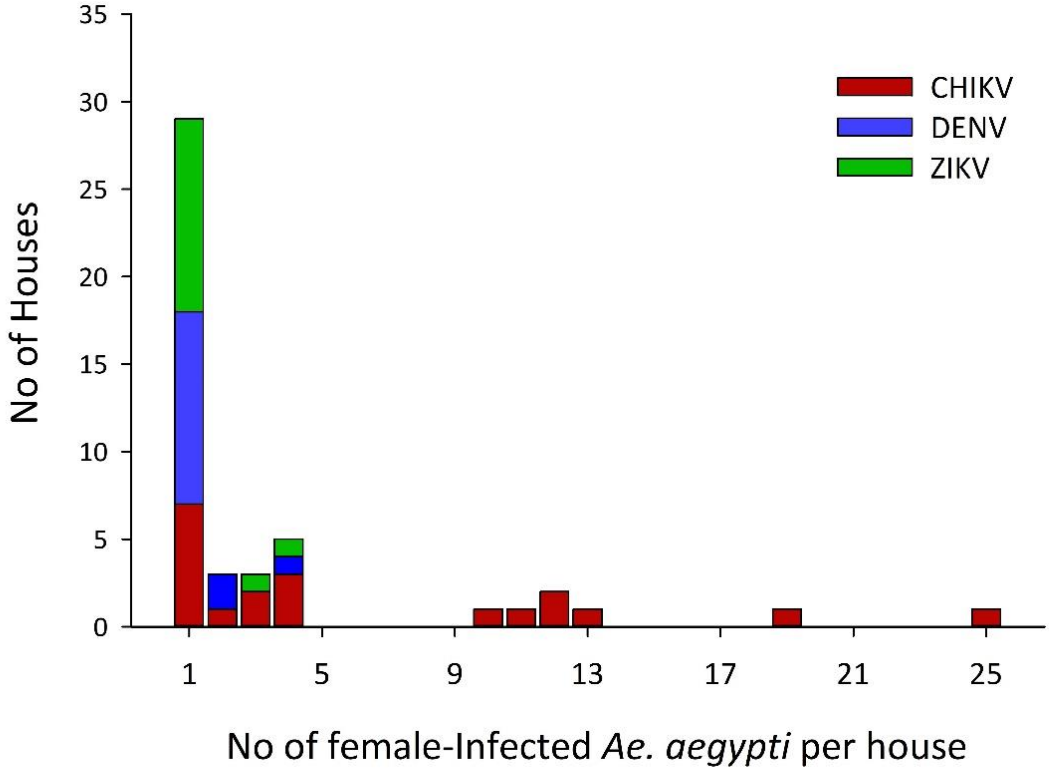

Fig. 2. Percentage of houses infested with female *Ae. aegypti* positive for any of the three targeted viruses in low-density (<10 total mosquitos per house, n=98) and high-density (> 10 total mosquitoes per house, n=70) premises. Panel A shows houses with positive bodies and heads and panel B shows the percentage of houses where only heads were positive. The variable co-occurrence contains percentages of houses where mosquitoes where positive for either virus within the same house, including 3 positive mosquitoes with coinfection between CHIKV and ZIKV.

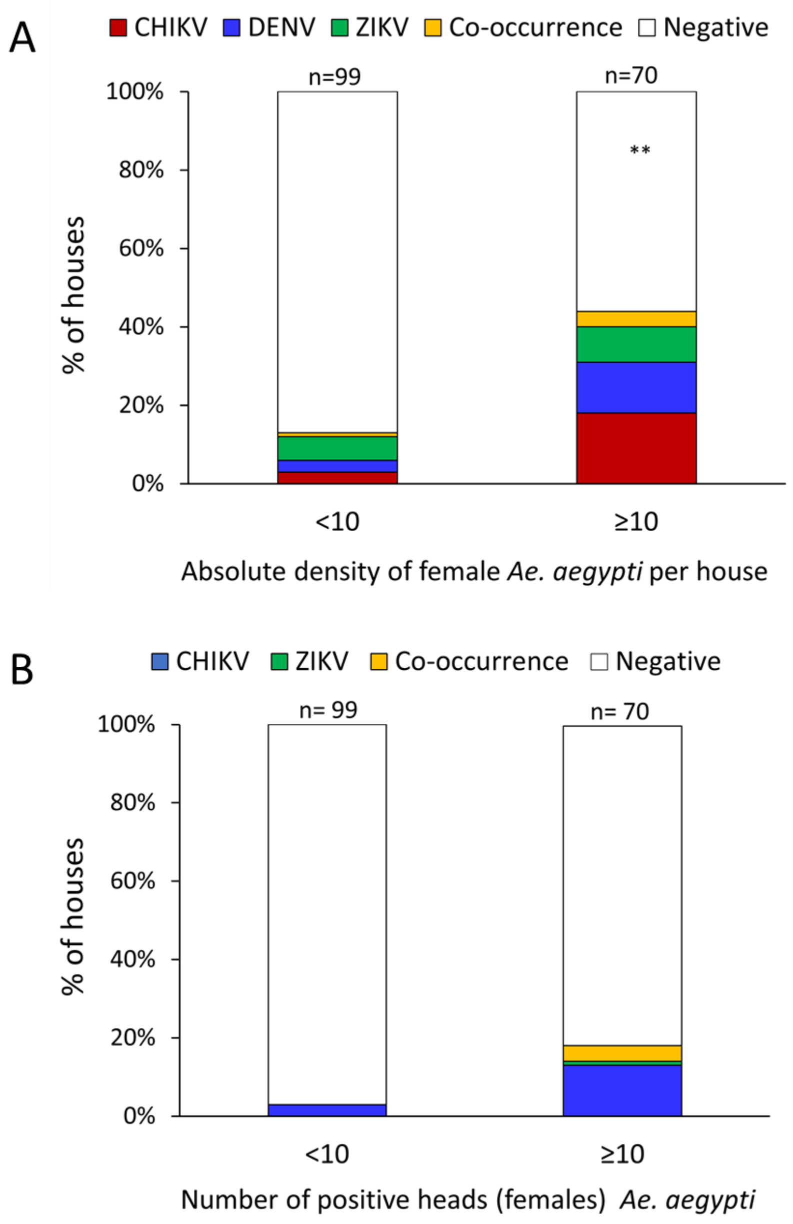

Fig. 3. Probability of detecting an infected female *Ae. aegypti* as a function of the total Ae. aegypti catch per house with evidence of recent arbovirus human infection. Solid line represents the mean prediction from a binomial generalized linear mixed effects model and gray band the 95% CI of the prediction.

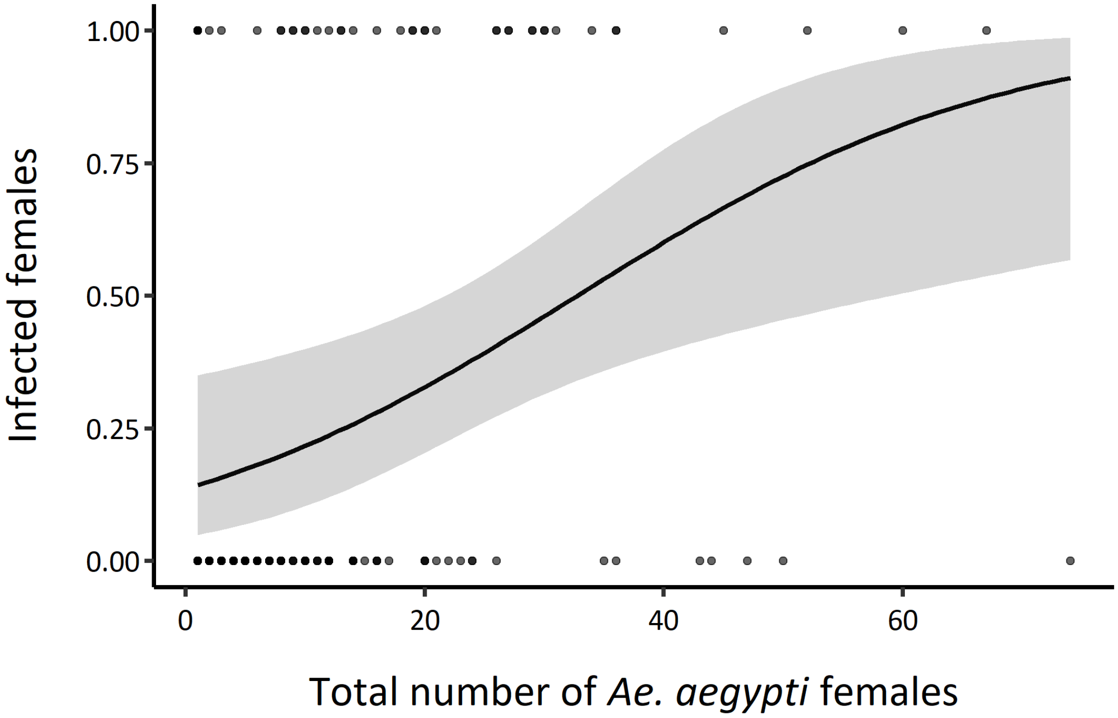

Fig. 4 – Cumulative probability of detecting houses with positive female *Ae. aegypti* (body and head) (A) and cumulative probability of detecting positive female *Ae. aegypti* (body and head) (B) for Chikungunya (CHIKV), Dengue (DENV) and/or Zika (ZIKV) in house as the collection effort increases in 10-min intervals.

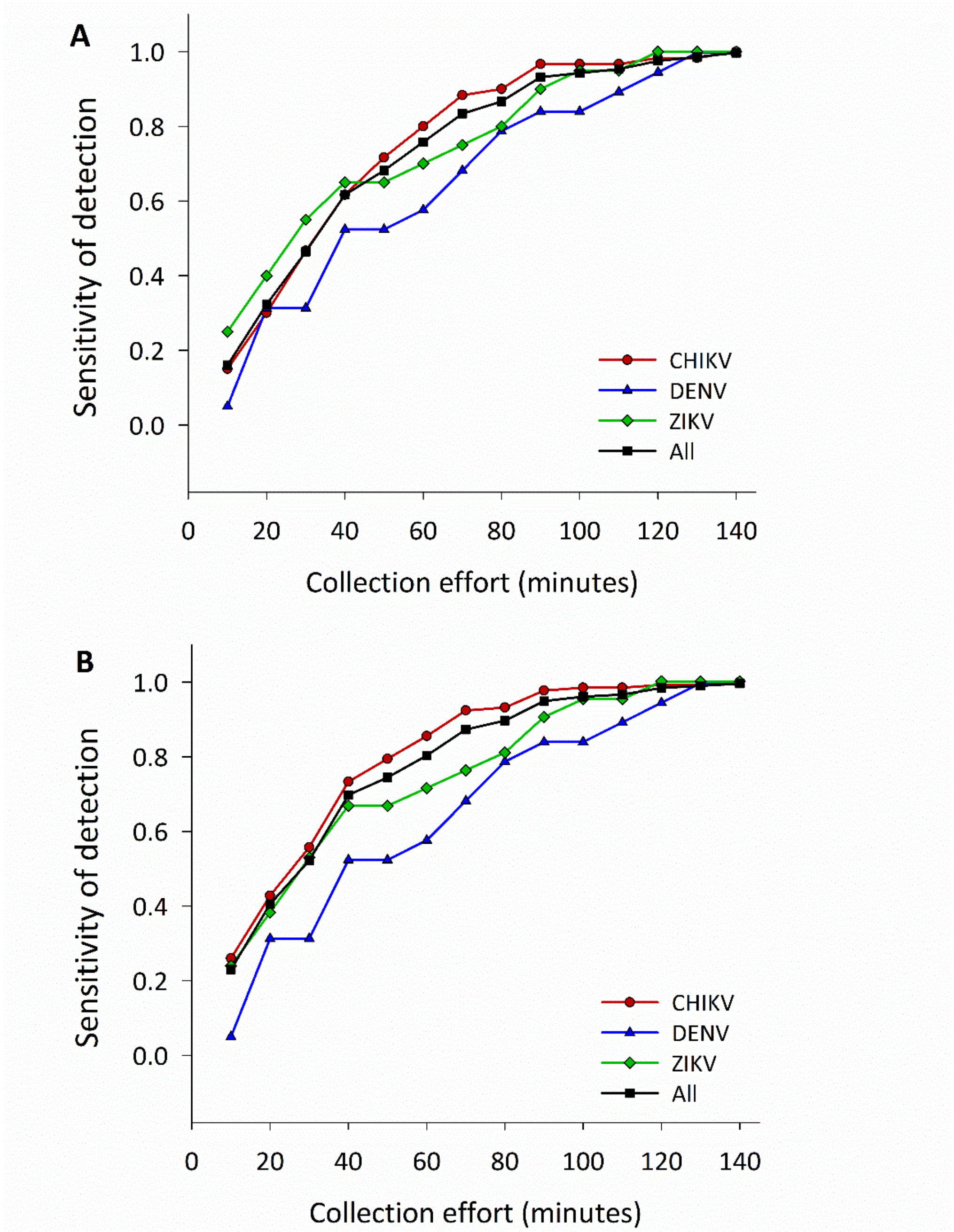

Fig. 5 - Household-level estimates of ABV transmission potential. The proportion of vectors per host (*m*), entomologic inoculation rate (EIR) and vectorial capacity (VC) were calculated per house and used to compare estimated between sample and total *Ae. aegypti* collections (panels A, C, E). Panels B, D and F show the association between total *Ae. aegypti* female abundance per house, and estimates of *m*, EIR and VC, respectively. Lines show the fit and confidence interval of a generalized-linear mixed model fitted to the data (Table S3).

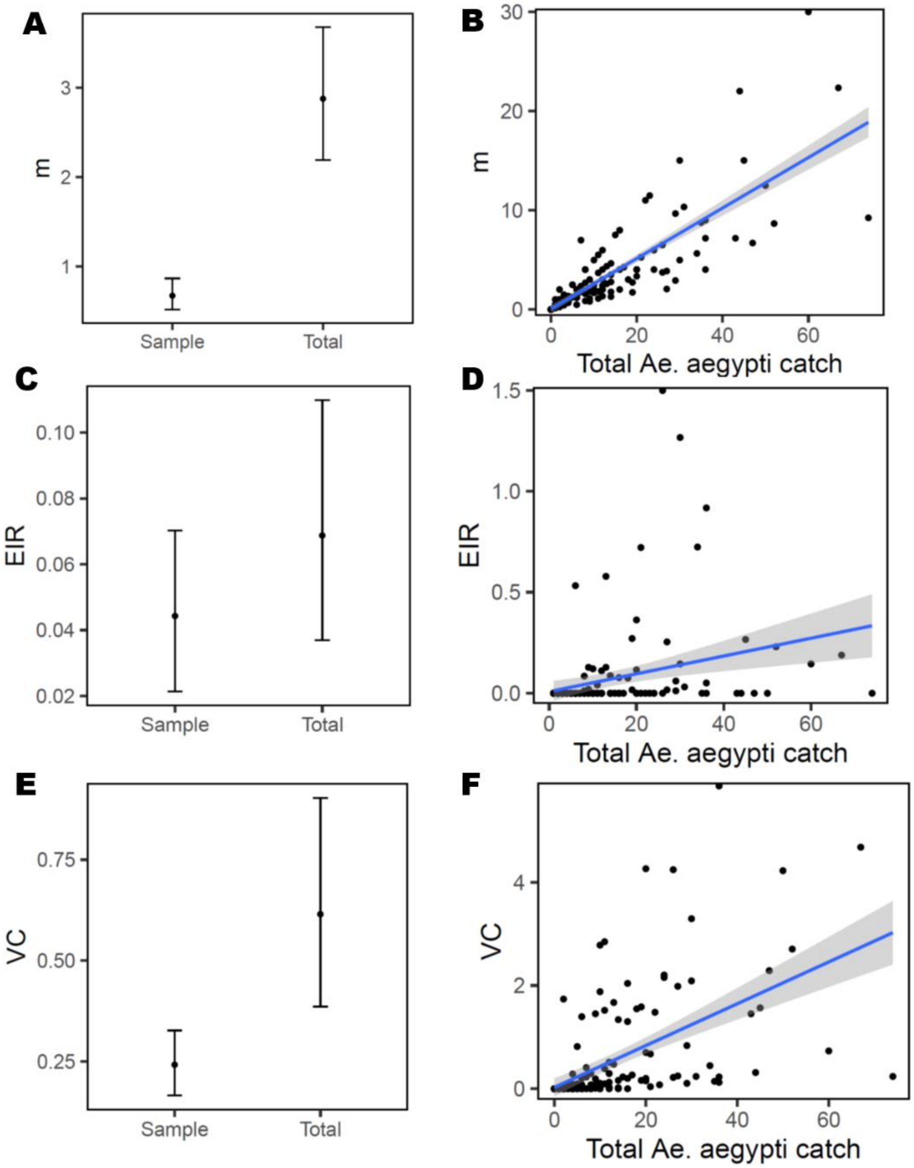

Supporting Information

Table S1 - Description and characteristics of real-time RT-PCR primer/probe sets used to target CHIK, ZIKV and DENV virus

Primer	Sequence	Probe	Working Concentration (µM)
panDENf	AAGGACTAGAGGTTAKAGGAGACCC		25
panDENr	CGYTCTGTGCCTGGAWTGATG	Quasar670 (Cy5)-BHQ2	25
panDENp	AACAGCATATTGACGCTGGGAIAGACCAG		10
CHIKf6856	TCACTCCCTGTTGGACTTGATAGA		25
CHIKr 6981	TTGACGAACAGAGTTAGGAACATACC	TAMRA-BHQ2	25
CHIKp 6919	AGGTACGCGCTTCAAGTTCGGCG		10
Zika1087f	CCGCTGCCCAACACAAG		25
Zika1163cr	CCACTAACGTTCTTTTGCAGACAT	FAM-BHQ1	25
Zika1108p	AGCCTACCTTGACAAGCAGTCAGACACTCAA		10

Table S2. Odd-ratio and 95% CI of the relationship between Sella scores and positive heads or positive bodies in *Ae. aegypti* collected from Yucatan, Mexico. No statistically significance was detected.

	**Infection in Bodies**			**Infection in Heads**		
	**OR**	**2.5%**	**97.5%**	**OR**	**2.50%**	**97.50%**
**Sella 1**	0.0	0.0	0.0	0.0	0.0	0.0
**Sella 2**	1.3	0.7	2.5	1.5	0.5	4.4
**Sella 3**	2.2	1.0	5.4	1.6	0.4	6.4
**Sella 4**	1.1	0.5	2.5	0.8	1.0	3.7
**Sella 5**	1.3	0.5	3.5	0.6	0.1	6.1
**Sella 6**	1.0	0.4	2.4	1.2	0.3	4.9
**Sella 7**	0.8	0.9	1.8	0.7	0.9	2.5

Table S3 - Model fits for the association between entomologic inoculation rate (**EIR**) or vectorial capacity (**VR**) and total catch of female *Ae. aegypti* indoors.

Model	Parameter	Estimate	SE	t	P
**EIR~Total catch**	Intercept	0.0379	0.0232	1.629	0.106
	Total catch	0.0020	0.0009	2.181	0.031
**VC~Total catch**	Intercept	-0.3323	0.1050	-3.166	0.001
	Total catch	0.0744	0.0046	16.155	< 2e-16

Table S4. List of arbovirus-positive sequences that were used to confirm infection in collected mosquitoes from Yucatan, Mexico.

Virus	Samples Pos	Seq Samples	Blast Samples	
CHIKV	129	100	92	76 Consensus
				16 Singlets
DENV^	19	17	12	3 Consensus
				9 Singlets post t
ZIKV	18	17	17	7 Consensus
				10 Singlets
	-->	7 heads*	7	5 Consensus
				2 Singlets
	-->	3 co-CHIKV	3	3 Consensus

* ZIKV heads matches with positive bodies.
^ DENV serotype corresponded to DENV-4

Fig. S1 – Number of clinical confirmed cases between 2015 and 2018 in Merida, Yucatan. Mexico. Data was obtained from SINAVE database, number of cases caused by CHIKV in 2015 was obtained from Mendes et al. 2018 [70]. Axis Y (Number of confirmed cases) is presented in Logarithmic scale.

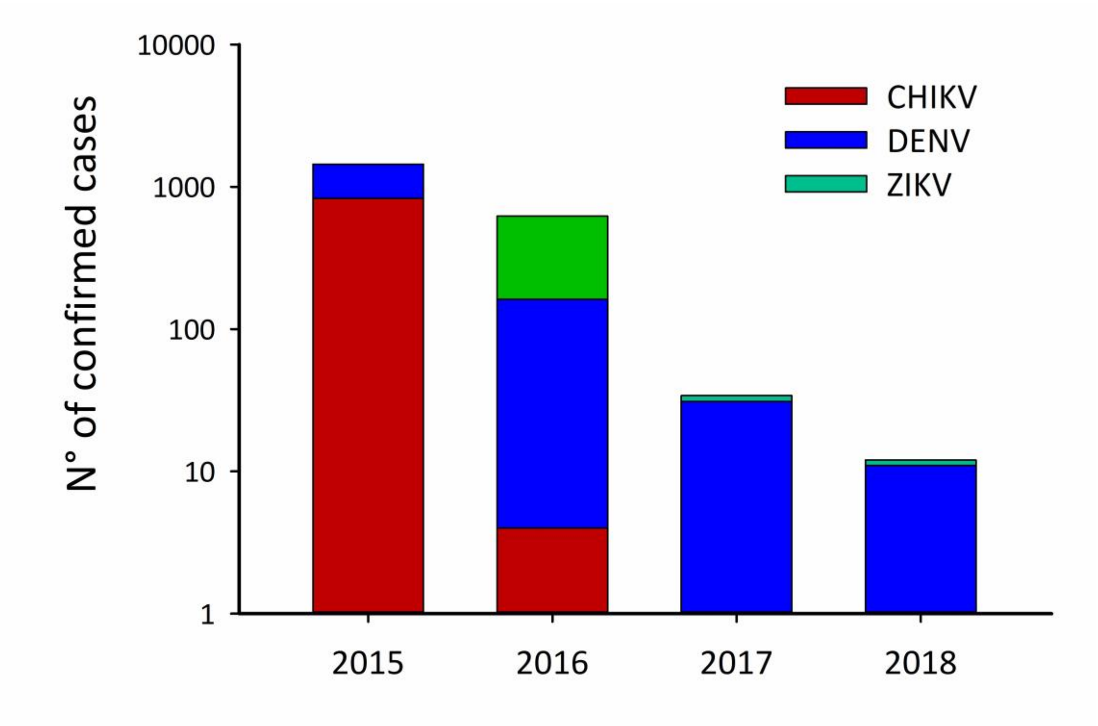

Fig. S2 - Distribution of human biting rate (*a*) by house.

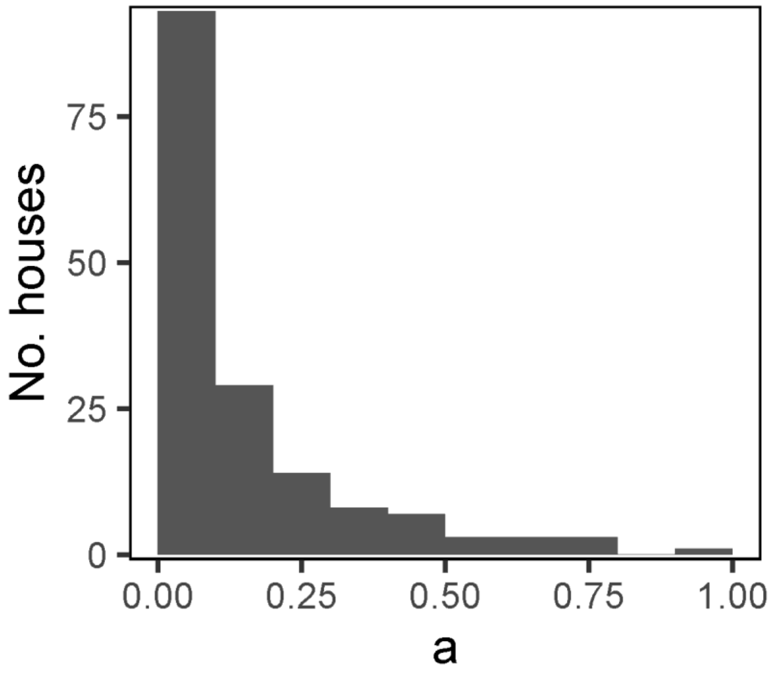

Supplement: Attachment — Submitted filename: PNTD-D-20-01322_reviewer (1) arbovirus detection .pdf [file pntd.0008972.s008.pdf]
