# Supplementary material for: Natural arbovirus infection rate and detectability of indoor female Aedes aegypti from Mérida, Yucatán, Mexico
Source: PLoS Negl Trop Dis. 2021 Jan 4;15(1):e0008972. doi: 10.1371/journal.pntd.0008972 (PMC7781390; doi:10.1371/journal.pntd.0008972)
Supplement: Attachment — Submitted filename: PLOSNTD_review_Sept1_2020.docx [file pntd.0008972.s009.docx]

**Review for “Natural arbovirus infection rate and detectability of indoor female *Aedes aegypti* from Merida, Yucatan, Mexico”**

This study focuses on quantifying *Aedes aegypti* density, and how sampling effort and mosquito density affect arbovirus detection rates, as well as estimates for arbovirus infection prevalence in sampled mosquitoes, the entomological inoculation rate, and overall vectorial capacity. This study provides a general approach and important data on the infection rates of indoor resting / questing mosquitoes that could be useful in targeting mosquito control and disease mitigation efforts. However, I have several major concerns that need to be addressed before this manuscript can be accepted for publication.

**Major Concerns:**

1. Introduction: I think the last paragraph needs to include a succinct summary of the questions this study is interested in addressing. This is particularly important for understanding the specific study design and evaluating whether it is appropriate for the questions the authors are addressing. It is also important for understanding the response variables the authors have focused, the downstream comparisons made, and the choice of specific statistical analyses. From the information provided, I am assuming that the authors are trying to estimate how the probability of detection of arbovirus in mosquitoes within a home is affected by mosquito densities within the home and sampling effort given a high probability that arbovirus is present in that home. Right?
2. Methods:
   a. More information is needed in the study design. How were specific houses identified for sampling? Was it solely based on infection status of household members or did the authors try to randomize across households that had a history of symptomatic infection? Also, why only focus on households that had a history of symptomatic infection (this might become clearer when the study questions are added)? How were these homes distributed across space and time? Did you select homes to control for inter-home correlation by choosing homes that were spaced a certain distance apart? How many times were homes sampled (once vs. repeatedly)? I realize that these methods are likely associated with the reference provided in the text, but it would be nice to have some of this here to contextualize the discussion of the results.
   b. I am unclear how the authors can justify that they are quantifying absolute vs. relative abundance of mosquitoes nor why this is an important distinction to make for the questions they are interested in addressing. To me relative abundance is absolute abundance (true abundance) multiplied by some estimate of capture efficiency. One can estimate absolute abundance by adjusting their measures of relative abundance by some imperfect detection rate. This has not been done in this study as currently written. In the current calculation of absolute abundance (sum aegypti over all of the 10 min intervals spanning 3 hr), the authors are assuming they have captured all of the aegypti in a given household. To me, this is just another measure of relative abundance (albeit likely a better one than just one 10 min interval) and if the authors want to get at absolute abundance than they need to adjust relative abundance by some metric of capture efficiency (which will go down with increasing effort). The authors may be able to get at this through the relationship between capture rate and time. If the authors do not want to do this, then they need to make their assumption that they have captured all of the aegypti in the house explicit in the manuscript and also discuss the implications for the interpretation of their results if this assumption is not upheld.
   c. Statistical analyses: I imagine that the ability to detect arbovirus infection in mosquitoes will not only depend on mosquito densities within the house but also the number of household members that were symptomatic. Do you have this information to include in your model analysis? Also, all of the models constructed and the associated AIC scores should be included somewhere in the manuscript along with other metrics of model performance (residual analysis, etc.). Finally, I am not an expert on Bayesian approaches, but maybe these approaches would be appropriate for these data that explore, given a conditional probability that the household has arbovirus, the probability of detecting that arbovirus in mosquitoes and what factors (e.g. sampling effort and mosquito density) influence that probability?
   d. Estimates of risk: Why use both EIR and VC? For the questions being addressed in this study as well as the implications for using data like these for targeted control, EIR seems a more relevant metric of risk. Further, the parameters comprising EIR can be directly estimated from the data generated in this study vs. VC, which has parameters that this study did not directly measure and likely change based on environmental conditions (mosquito survival rate and the extrinsic incubation period). Also, why is the probability of a mosquito becoming infected after biting an infectious person not included? This formulation of VC also has dimension issues. See: Massad Eduardo, Coutinho Francisco Antonio Bezerra. Vectorial capacity, basic reproduction number, force of infection and all that: formal notation to complete and adjust their classical concepts and equations. Mem. Inst. Oswaldo Cruz. 2012;  107 (4): 564-567. <https://doi.org/10.1590/S0074-02762012000400022>. I would recommend dropping VC unless further justification is provided and the potential limitations of VC are acknowledged in the discussion section.
3. Discussion:
   a. In the introduction and the discussion section, the authors make statements about the sensitivity of the Prokopack aspirators in detecting arbovirus infected mosquitoes and how this probability detection will be dependent on collection method. I would make sure that the language here is careful and reflects what the study actually examined. As currently reads, it seems as if the authors are arguing that the Prokopack is more sensitive to other collection methods (CDC backpack aspirator and other trapping methods). While this might be true, this is not what the study actually set out to measure. So clarify the language throughout to reflect how the factors measured (e.g. mosquito density, amount of effort, number of blood-fed mosquitoes, etc.) affect the sensitivity of detecting arbovirus infected mosquitoes.
   b. I think there are alternative (but not mutually exclusive) hypotheses that explain the overdispersion of infected mosquitoes across houses sampled. The aggregation of mosquito bites on a few attractive hosts could potentially explain this, but you are then assuming that mosquitoes can actively select certain households. As mentioned by the authors, aegypti does not disperse very far, so depending on how the sampled households in this study are located across space, there may be other explanations for the overdispersion. For example, variation in housing across the city could affect the accessibility of indoor habitat for questing and resting females, which would then affect the overall mosquito densities in a house. Further, what factors drive spatial variation in human arbovirus infections? Where the relatively few households that generated the most arbovirus infections in mosquitoes connected in any way or located spatially in a similar area? A broader discussion on potential mechanisms here is warranted.
   c. I would add a concluding paragraph that summarizes the key implications the authors would like the readers to walk away with

**Minor Concerns:**

Line 39-40: Ae. aegypti needs to be italicized

Line 87-91: consider combining these two sentences with the following paragraph. Generally, for a paragraph to stand alone, there needs to be a minimum of three sentences. I also think these two sentences thematically align with the information in the following paragraph.

Line 114: “was detected 10 days...” in what? I am assuming field collected mosquitoes?

Line 148: (within 1 month) should be (within one month) – generally all numbers from 1-9 should be written out unless at the beginning of a sentence. I would go through the entire manuscript for this one, as there are multiple instances where this needs to be corrected.

Line 234: Based on the topic sentence of this paragraph (lines 231-232), VC and its definition should follow EIR and its definition before getting into the specifics of each metric.

Line 244: n = extrinsic incubation period should be the number of days on average it takes for the mosquito to become infectious (so, I am assuming this is 5 days). As currently written, this is defined as the extrinsic incubation rate (1/5 days), which would be incorrect.

Line 257-258: “Of the total ABV-infected females, 38 (22.9%) had evidence of infection in their heads; 33 (86.8%) of them were positive for CHIKV...” Do the authors mean 33 of the total ABV-infected females or 33 of the ABV-infected females with positive heads?

In general, the authors should make it really clear how the percentages presented throughout this manuscript are calculated. Especially in the associated tables (1-3), which should stand alone from the text, it is completely unclear what the percentages in parentheses are referring to.

Line 272: “follow” should read “follows”

Line 281: “When mosquito density was high…” I would clarify that this is “When within household mosquito density was high…”

Line 340: “considered a gold standard for indoor adult Ae. aegypti collections” – While this might be the case, I do not think this statement fits with what this sentence is trying to convey and I would recommend removing it. Especially since this study is not comparing sensitivity across different collection methods, this is confusing.

Line 345: “will be a function of the collection method used” – I would provide a reference here to support this statement as this study did not actually measure this.

Line 348-350: “These data show that the low apparent…may also be a function of the sensitivity of the collection method.” I would clarify this sentence to reflect what this study actually measured – again, this study did not demonstrate the effectiveness of the Prokopack method relative to other collection methods, so starting the sentence with “these data show…” is somewhat misleading.

Line 355-357: “we evidenced its powerful epidemiological effect in the strong overdispersion of infection in collected mosquitoes.” I am not sure what the authors really are saying here. Please clarify. Also, can you really say that your data demonstrate heterogeneous biting? Or rather, that some households had more mosquitoes than others and the distribution of households with high infection rates also had high mosquito densities, so something to do with variation in housing infrastructure and mosquito habitat? I agree that people are not equally attractive, but I think other factors are likely driving the distribution of infected mosquitoes.

Line 358-359: see above comment. Also, do you know how many members of each household sampled were symptomatic for arbovirus infection?

Line 369: this sentence is not as clear as it could be. I suggest: “Wolbachia population replacement or suppression approaches rely on the release of Wolbachia infected adults, which when mated to wildtype, uninfected adults render the population incompetent to transmission or reduce adult female density, respectively.”

Line 372-376: This sentence needs clarification and this paragraph needs a better topic statement - I think I know what you are trying to say here...that the effective deployment and assessment of these technologies require good estimates of mosquito distributions across households, within household mosquito densities, and arbovirus infection rates. You should lead with this and then provide examples demonstrating why this is important.

Line 389: “bias” should be “to bias”

Line 393-396: The study design of the referenced study was different from the one in this study. It appears from the referenced study that they did not target efforts to households with confirmed symptomatic infection in household members. So, the prevalence would naturally be much lower than this study I would expect. So, I don’t understand the point the authors are trying to make here.

Line 406: “This data aligns” should be “These data align”

Line 413: “thought” should be “through”

Liine 418-419: “DENV-4 the predominated in the most years” is awkward. Consider “This result lines up with previous results obtained from different work in the area where all four DENV serotypes were found circulating in Merida, with DENV-4 being the predominant serotype in most years along with DENV-1 and DENV-3 serotypes.”

Line 424-426: Well...there is the salivary gland barrier that needs to be breached and can be a bottleneck in the infection process...so just because you see it in the head does not mean it is in the saliva...I would be careful here - maybe acknowledge this and then state that it is likely that these mosquitoes are infectious.

Line 435: I would argue that VC in this sense is not as good as EIR because multiple parameters you assume values for and these will be sensitive to environmental conditions.

Table 1: from the table legend, it is unclear how the percentages in the parentheses are calculated.

Table 2 figure legend “either” ??? remove? Also, the are the percentages in this table reflecting the number of mosquitoes with positive heads out of total number of mosquitoes infected? Captured?

Table 3: again, how are the percentages calculated – tables and figures need to stand alone. I am assuming these are the number of mosquitoes with positive heads divided by the number of mosquitoes captured with each Sella score?

Figure 2 – maintain the same colors for each virus across each panel A and B – also line 697, 3 should be three

Figure 3 – the y-axis is confusing…it reads like the number, when this is really a probability of detection of infected females

Figure 4 – what is the difference between A and B?

Figure 5 – sample vs. total – do you mean first 10 min interval vs. the sum total across all 10 min intervals?
