# Supplementary material for: Natural arbovirus infection rate and detectability of indoor female Aedes aegypti from Mérida, Yucatán, Mexico
Source: PLoS Negl Trop Dis. 2021 Jan 4;15(1):e0008972. doi: 10.1371/journal.pntd.0008972 (PMC7781390; doi:10.1371/journal.pntd.0008972)
Supplement: Attachment — Submitted filename: Kirstein_etal.2020_RebuttalUpdated.docx [file pntd.0008972.s010.docx]

**Rebuttal**

We thank the three anonymous reviewers for their evaluation and suggestions for improvement of our manuscript. We have addressed them in the point-by-point response to reviews provided below. We organized the responses by reviewer, rather than section, as reviewers 1 and 2 provided minor suggestions, and reviewer 3 had extensive comments that were repeated in multiple sections, making the section-by-section review quite confusing. We do keep the section titles to guide each reviewer to their original comment. Reviewer’s comments are presented in italics, while our responses are presented with bold font.

***Reviewer #1.***

***Summary and General Comments***

***Reviewer #1:*** *This paper describes surveillance inside houses for Aedes born viruses in Merida Mexico. In particular it provides excellent quantitative data on the incidence of viremia mosquitoes in particular CHIKV, ZIKAV and DENV infections during concurrent outbreaks of these diseases. This is a very welcome addition to the literature. This data is then used to calculate indices of vectorial capacity for this important arbovirus vector. I think this is a very significant piece of research and should be published.*

**R. We thank the reviewer for the positive evaluation.**

*I do have a few comments that I would like the authors to consider and hopefully will improve the quality of the manuscript. While the use of aspirator collections particularly to collect mosquitoes indoors is a gold standard, there are some practical considerations that the authors must consider. In particular, during outbreaks of the coronavirus, it is all but impossible to enter people’s premises. While this hopefully it is but a temporary issue (I hope!), I still think it warrants I mentioned by the authors.*

**R. We agree COVID-19 has changed the way vector surveillance and control are conducted in endemic countries. We also hope it is temporary and include a sentence in the discussion section outlining the challenges of entomological surveillance indoors. Our current operations as part of a large CRCT have been delayed. Traps may fill an important gap. However, we argue that studies like ours should also be conducted with traps, to quantify their sensitivity and correlation with vector density. L454 - 459 of the Discussion section now reads as this: “Furthermore, in the context of the current COVID-19 outbreak, methods such as indoor adult mosquito aspiration may not be easily implemented due to difficulties personnel my encounter in gaining access to the home environment. Aedes collection traps (e.g., GAT, BG sentinel trap, sticky ovitrap) would provide a viable alternative to indoor aspiration. Based on our findings we argue that future research should focus on calibrating such trapping methods to assess their sensitivity to detect Ae. aegypti and ABV-infected female mosquitoes.”**

*Also, some of the practical considerations of having to do with repeated collections within a house in order to detect aren’t there ways that this could be optimized?  Other sampling methods could be used at least to provide information on virus incidence in the mosquito population, and to measure the impact of a large-scale intervention such as TIRS. This would include use of gravid Aedes traps to capture adult females which has been done in several other studies.*

**R. We agree with the reviewer that multiple methods to quantify *Ae. aegypti* (indoors and outdoors) exist. Here we focused on ‘calibrating’ the Prokopack collections to the detectability of infected mosquitoes. We do not expect our sequential sampling to be adequate for quantifying mosquito infection. Rather, we see it as an opportunity to calibrate a method that is based on a catch-per-unit effort. However, we take the point made by the reviewer about being flexible due to COVID-19, and in the context of the TIRS study we will be considering GATs if indoor access is limited in the coming years.**

*Finally, I think you should discuss the fact that Ae. aegypti is known to take multiple feeding and indeed biting attempts to obtain a bloodmeal.  How does this affect VC/EIR?*

**R. We mention heterogeneous biting in the discussion, and now extend our interpretation of entomological metrics under the context of multiple feeds on the same host.**  *Discuss implications of PCR antigen vs live virus detection.*

**R. We included in the discussion the fact we could not conduct virus isolation, and the validity of our findings despite such caveat. L515 – 522 of the discussion now reads: “While PCR is a mainstream method for virus detection, ultimate confirmation of virus infection in mosquitoes should be done through virus isolation techniques using cell culture/suckling mice. Unfortunately, our field laboratory did not have the required BSL level and approvals to conduct virus isolation, as CHIKV requires a BSL3 facility. Despite this limitation, we consider our findings robust and tractable, because: a)** **sequencing of most PCR+ samples led to a direct match with the virus detected by PCR; and b) we found a 100% match between positive heads and positive bodies, indicating PCR conducted on the same individual led to similar results.”**

*I think this is a really nice piece of work and it’s great to see some actual field collections including multiple infections of viruses within Ae. aegypti. Well done.*

**R***.* **Thank you.**  *Also please see other comments on the attached PDF.*

**R. Addressed and considered.**

***Methods***

*Yes, but what about PPE for sampling staff?*

**R. Staff used regular field clothes, which included closed toes shoes, socks, pants, and long-sleeve shirt. No DEET was used as this impact’s mosquito behavior. No illness was reported on any of our field staff. The study was conducted prior to COVID-19, so no specific PPE was required. We added such description to the methods section (L177)**

***Results***

*Does the analysis presented match the analysis plan? YES*

**R. Thank you.**

***Conclusions***

*Maybe consider alternative methods for measuring mosquito infections especially in COVID...Also, practicality of aspirating for large scale monitoring*
**R. The discussion now includes a mention of alternative methods in the context of COVID-19.**

***Editorial and Data Presentation Modifications?***

*Minor*

***Reviewer #2***

***General Comments***

*The authors published a study in 2019 estimating absolute indoor density of Aedes aegypti using sequential sampling of aspirators.  This current study now builds on that prior work by reporting the virus testing results for these mosquitoes sampled from households with positive human cases as well as using these data to estimate the entomological inoculation rate and vectorial capacity.  These are very unique data, and the authors executed a very challenging study to gather these data. I’ll present a few discussion points below in hope of improving the manuscript.*
**R. We appreciate the positive response by the reviewer.**

*These infection levels appear abnormally high.  It would be helpful for the discussion to compare these results to other studies.  Obviously, this current student was targeting homes within a month of symptomatic humans infected with one of the three viruses.  Virus infection studies in Ae. aegypti tend to report very low infection rates, especially for surveillance traps unrelated to locations of human cases, which is hard to compare to the current study*

*In your case you present an infection prevalence of 3.8% of your individual female Ae. aegypti being positive for CHIKV. The paper below is a good example of a study targeting Ae. aegypti collections by aspirators from inside the homes of human patients and they still only had 6 of 644 mosquitoes positive (0.9%).  The current study appears to have very high infection levels in the collected mosquitoes so more explanation of this would help.
Anders KL, Nga LH, Thuy NTV, Ngoc TV, Tam CT, Tai LTH, et al. (2015) Households as Foci for Dengue Transmission in Highly Urban Vietnam. PLoS Negl Trop Dis 9(2): e0003528. doi:10.1371/journal. pntd.0003528*
**R. We conducted collections in a period post-invasion of Chikungunya and early invasion of Zika, which means that our overall infection rate of 7.7% includes all three viruses and not only dengue. If we calculate the infection rate for dengue, we obtain 19+mosquitoes / 2161 collected = 0.87% infection rate. This rate is not different from the 0.9% reported by Anders. The interesting finding from our study is that, when multiple viruses co-circulate and transmit epidemically in different years, infection rates may be influenced by the dominant virus. This dynamic may be different between Latin America and SE Asia, as large outbreaks of the three viruses are more** **prevalent in the former.** **We now make reference to the Anders et al. paper and explain more clearly the similarities between studies with regards to DENV infection. L474 – 490 in the discussion incorporate this observation: “Information from individually tested mosquitoes is more reliable, yet few studies have undertaking such expensive and time-consuming task. I In Mérida, RNA extracted from individual Ae. aegypti females collected in periods of high and low arbovirus transmission and tested by RT-PCR [30]led to estimates of DENV natural infection rate of <1% [30]. A similarly low DENV natural infection rate from individual mosquitoes was quantified both in Yogyakarta, Indonesia [62] and, in Ho Chi Minh City, Vietnam [63]. Particularly for DENV, our study found a similarly low natural infection rate (19/2,161 = 0.87%), in agreement with previous reports and consistent with the low number of reported dengue cases in the city (Sup. Fig 1). Unlike such reports, which only focused on DENV, our study was conducted in the context of ongoing dengue transmission, and during CHIKV and ZIKV invasion of Mérida. Most of the positive Ae. aegypti had evidence of CHIKV infection during the first year of collection, which represented the second year post-CHIKV invasion in Mérida, leading this virus to contribute with three quarters of all infected mosquitoes to the overall 7.7% ABV natural infection rate. Our findings clearly show that, when multiple viruses co-circulate and transmit epidemically, infection rates may be influenced by the dominant virus. Surprisingly, in our study the high prevalence of infection by CHIKV in Ae. aegypti occurred in in 2016 during the emergence of ZIKA and a period when the most reported infections in Mérida were due to DENV (Sup. Fig 1). “**

*Figure S1 is very valuable as this understanding of virus circulation in the human populations at the community level is necessary to help interpret these mosquito infection results. The figure shows the total number of cases in 2015-2018.  I think line 150 shows that the surveillance of cases and mosquito sampling occurred in 2016-2017.  It would help if the methods could clarify when mosquitoes were sampled, including the months of collection.  The tables and figures (e.g. Table 1, Fig. 1), could also include the year of collection in the table legend just for clarity.*

**R. We now include year and month on each figure and legend. Additionally, Table 1 now separate estimates by collection year.**

*As I review the results, it seems as though all the data are grouped together ignoring the temporal component of when the samples were collected.  This is unfortunate because the Fig. S1 shows very high variation in the amount of human cases for each virus in each year.  A way to help resolve this would be if Table 1 could go from a single column of data as currently written to three columns (e.g. 2016, 2017, Total). The justification for paying more attention to when the samples were obtained is that given that 3/4ths of the positive pools were with CHIKV, we would assume most of those were from earlier in the sampling compared to later.  In the discussion you say CHIKV was found in mosquitoes when most reported human infection was ZIKV.  This further emphasizes the need to better present these data.*
**R. We agree with this good point and observation by the reviewer, and Table 1 now includes three columns as suggested. Indeed, findings align in that most CHIKV+ mosquitoes were collected in 2016 and most DENV+ in 2017. Thank you for your suggestion.**

*I also predict that comparing the high CHIKV in the mosquitoes there will still be a lack of correlation to what the SINAVE database reported for the community of Merida, Yucatan.  Meaning the vast majority of human cases in 2016-2017 were not CHIKV so it is puzzling how this study documented so many CHIKV infections in mosquitoes.  Your discussion trys to explain this but to help resolve, another improvement to this study would be to incorporate which virus was responsible for the human cases in the homes that were sampled.  The methods explain that the Ministry of Health provided the addresses for human cases which was then sampled within 1 month of the patients being symptomatic.  Is it possible to obtain the additional details regarding which homes had which viruses present when the mosquitoes were collected?  You already know it was one of three viruses so knowing which virus specifically would likely avoid IRB issues with humans’ subjects (plus IRB already reviewed this study).  While presenting all these mosquito infection data it would be much more valuable to know which virus was in the occupants of the household.  For example, a virus that matches the mosquito and the household would suggest the occupants of the household resulted in exposure.  Alternatively, if they don’t match, it could suggest visitors to the home could have been infected.  This would especially be interesting to know for the mosquitoes co-infected with 2 viruses.*

**R. As we noted, and the reviewer rightly points out, having data from infection status of each household could help address the mismatch between infection and reported cases. Unfortunately, we have no access to such information, which was provided by Yucatan MOH and not the FSD cohort. As reported, our FSD detected few symptomatic cases in 2015 (8 ZIKV, 12 DENV, 30 CHIKV; Rojas et al. 2018 .PLoS Negl Trop Dis 12(11):e0006847) leading us to focus on passive surveillance data collected by the Yucatan MOH to achieve our target of 200 houses during 2016-2017. Such dataset was not included in the FSD IRB and the level of masking in the data was determined by Yucatan MOH. While Yucatan MOH provided information for each virus geocoded to the census tract level (Bisanzio et al. PLoS NTD 2018), we could not obtain information that could identify which virus each household was positive to. We just managed to get the list of houses that were positive. We made clearer in the methods this limitation and, as reviewer 2, we also regret to have this unfortunate gap in our dataset.**
 *Were vector control activities happening during the course of this study?  That would help to know if these data generated here are during epidemics with no control, moderate control, intensive control, etc.  The sampling focused on indoor populations of Ae. aegypti so I assume sampling of outdoor populations would have been different, especially in the presence of outdoor control efforts.*

**R. Vector control activities occurred routinely in Merida during the study. These involve primarily truck-mounted ULV spraying of chlorpyrifos. While we do not have information of the specific routes the MOH trucks followed, there is plenty of evidence supporting the fact that their impact on indoor *Ae. aegypti* tends to be transient and very limited. Therefore, we agree with the reviewer that sampling outdoors is not only more difficult because *Ae. aegypti* are harder to find, but also it may be impacted by insecticide applications.**

*Table 3 is an interesting perspective looking at virus infection in bodies relative to heads for the positive individuals. It appears that 3% of the heads of unfed (parous and nulliparous) females with positive bodies were also infected.  Then 7.2% of the heads with a fresh blood meal were positive.  But what does not make sense is that you would expect that as an infected blood meal digests, the virus would disseminate in a portion and eventually reach the head.  So as the sSlla score increases, ending at a gravid female, you would expect the heads to have a higher percent infection than unfeds.  The percent infection of gravid females is 4.2% but the ones with a fresh blood meal less than a day old is 7.2%.  This leads me to believe that there is a chance the specimens with a fresh blood meal could have had contamination from the virus in the blood meal resulting in the head testing positive.  It would be good for the discussion to pay more attention to this.*

**R. Assuming we are sampling the same ‘cohort’ of adult mosquitoes, with the same age and emerged at the same time and bit a viremic person at the same moment , the mismatch pointed by the reviewer would be true. However, we could not determine the age of the mosquitoes and it is very likely we are seeing adults from different cohorts that could have gotten an infectious meal at different times. As we received information about infection in the houses, we visited them as soon as possible, but delays in the MOH reporting limited our ability to survey the house within the same week of the case being seen by a doctor. Given *Ae. aegypti* feeds very frequently (every 1.5-2 days), the finding of a high proportion of females with infected heads and fully engorged may be indicative of mosquitoes that incubated the virus and are feeding now for their 2^nd^ or 3^rd^ time after the initial viremic bloodmeal. Also, keep in mind we are sampling mosquitoes indoors, and some gravid (and potentially infected) females may likely leave the house to lay eggs outside. We include these observations now in L533 - 543 of the discussion: “Generally, mosquitoes are assumed to be infective when viral infection is detected in their head, which could indicate infection of the salivary glands. We found 38 female specimens with positive head, 86.8% of those were CHIKV-positive. Gravid females (Sella's score 7) with positive heads were also detected (7 specimens), but the majority of adults with infected heads had a Sella score of 2, indicative of mosquitoes that fed within the prior 24 h. As we are not sampling the same ‘cohort’ of adult mosquitoes, and visited the houses within one month of the report of a symptomatic case, we can hypothesize that those adults with positive heads acquired the viral infection from a viremic human, survived the extrinsic incubation period and just had a recent bloodmeal that likely led to virus inoculation on their human hosts. Quantifying the likelihood of such an important epidemiological event from our raw data would be very speculative, that’s why we used our results to innovatively calculate indices of transmission potential or risk (VC or EIR) not commonly estimated for ABVs.”**

***Methods***

*The methods are excellent with the exception of missing details outlined in the summary statements.*

**R. Outlined details were already considered and corrected.**

***Results***

*The results section is sufficient with the exception of suggested corrections in the summary statements.*
**R. Corrections were made.**

***Editorial and Data Presentation Modifications?***  *Minor comments:

Ln. 36.  Replace ‘any’ to ‘at least one’*

***R. We retain “any” since it refereeing to “any” of the viruses we targeted and not to the number of virus.***  *Ln. 40.  At this point in the abstract it is not clear how ‘total catch’ and ‘number sampled’ are different.  They sound like they are referring to the same thing but with different terms so try to better articulate the difference.*

**R. We edited the abstract for clarity, and now more clearly mention what ‘total catch’ refers to.**  *Ln. 42.  You present two percentages and then say ‘respectively’, but it is not clear what these percentages refer to.*

**R. The abstract was edited and corrected.**  *Ln. 59.  You use the phrase “entomo-virological approaches for virus surveillance”.  Why not just swap this out with ‘arbovirus surveillance’?*

**R. We changed it to “arbovirus surveillance in mosquitoes”.** *Ln. 68.  This statement sounds awkward: “vector surveillance via entomo-virologic surveys”.  Can you swap out with something like “virus testing of vector populations”.*

**R. Replaced***.

Ln. 89.  This additional citation would be appropriate for this statement:
Ramirez et al. 2018. Searching for the proverbial needle in a haystack: advances in mosquito-borne arbovirus surveillance. Parasit Vectors. 11: 320.*

**R. Considered and added.** *Ln 325.  I think ‘the’ should be replaced with ‘of’.*

**R. Considered and replaced.** *Ln. 357-359.  You are claiming aggregation of bites based on these data but as discussed above, you are not presenting any data on the human infections.  You could have had CHIKV pos mosquitoes in a home with a DEN patient.  Or you could have had many CHIKV pos mosquitoes in a home with all occupants being CHIK patents.*

**R. We agree with the reviewer that without information about the virus infecting the house, one can merely speculate about the likely explanation of the source of infection. We decided to indicate likely mechanisms in no order and point to the limitations in our study that prevented us from ultimately inferring the source of mosquito infections. L423 - 429 of the discussion now reads: “Seven houses harbored more than 10 CHIVK infected females each, with one having up to 25 infected females. Multiple mechanisms could have been responsible of such overdispersion, including aggregation of bites on one or a few infected individuals, mosquito biting on multiple viremic visitors to the house, or the dispersal of infected mosquitoes from nearby premises. Limitations in our study design prevented us from identifying whether those houses with aggregated infection in mosquitoes had also one or multiple infected humans, limiting our ability to accurately determine the factors responsible for the large number of infected mosquitoes.”**

*Ln. 391.  Check the papers below which might also provide DENV infection data for individual Ae. aegypti.
Rahayu et al. 2019. Prevalence and Distribution of Dengue Virus in Aedes aegypti in Yogyakarta City before Deployment of Wolbachia Infected Aedes aegypti. Int J Environ Res Public Health. 2019 May; 16(10): 1742.*

**R. Considered appropriated and added.**

*Anders KL, Nga LH, Thuy NTV, Ngoc TV, Tam CT, Tai LTH, et al. (2015) Households as Foci for Dengue Transmission in Highly Urban Vietnam. PLoS Negl Trop Dis 9(2): e0003528. doi:10.1371/journal. pntd.0003528*
**R. Considered appropriated and added.** *In several locations’ scientific names (e.g. Ae. aegypti) need to be italicized.*

**R. Done.** *Fig. 3.  Your legend could indicate what the dots at zero and 1 represent.  I assume those are the raw binomial data with a sample at a given number per house that tested either pos or negative.  But it looks like they have shades from light to dark and that is not explained.*

**R. The reviewer is correct, the points represent the binomial data. Darker colors occur because there is more than one point overlapping at that value of X-Y. We indicated that in the figure legend.**

***Reviewer #3***

***General Comments***

*This study focuses on quantifying Aedes aegypti density, and how sampling effort and mosquito density affect arbovirus detection rates, as well as estimates for arbovirus infection prevalence in sampled mosquitoes, the entomological inoculation rate, and overall vectorial capacity. This study provides a general approach and important data on the infection rates of indoor resting / questing mosquitoes that could be useful in targeting mosquito control and disease mitigation efforts. However, I have several major concerns that need to be addressed before this manuscript can be accepted for publication.*

***Major Concerns:
1.    Introduction:*** *I think the last paragraph needs to include a succinct summary of the questions this study is interested in addressing. This is particularly important for understanding the specific study design and evaluating whether it is appropriate for the questions the authors are addressing. It is also important for understanding the response variables the authors have focused, the downstream comparisons made, and the choice of specific statistical analyses. From the information provided, I am assuming that the authors are trying to estimate how the probability of detection of arbovirus in mosquitoes within a home is affected by mosquito densities within the home and sampling effort given a high probability that arbovirus is present in that home. Right?*

**R. We consider this not to be a major issue in our article, as it only involved minimal rewriting of the last sentence of the introduction to better describe the goals of the study. The rewritten section in L142 – 148 now reads: “Specifically, we quantified: a) how arbovirus infection in Ae. aegypti (overall and for each virus separately) is distributed across houses with suspected ABV transmission; b) the association between Ae. aegypti infection and different measures of indoor adult vector density; c) the sensitivity of indoor adult *Ae. aegypti* collections using Prokopack aspirators in detecting ABV-infected mosquitoes; d) effect of imperfect sampling of the adult population and vector density on two entomological measures of virus transmission potential, the entomological inoculation rate [43] and vectorial capacity [44].”**

***2.    Methods:*** *a.    More information is needed in the study design. How were specific houses identified for sampling? Was it solely based on infection status of household members or did the authors try to randomize across households that had a history of symptomatic infection? Also, why only focus on households that had a history of symptomatic infection (this might become clearer when the study questions are added)? How were these homes distributed across space and time? Did you select homes to control for inter-home correlation by choosing homes that were spaced a certain distance apart? How many times were homes sampled (once vs. repeatedly)? I realize that these methods are likely associated with the reference provided in the text, but it would be nice to have some of this here to contextualize the discussion of the results.*

**R. As explained to reviewer 2 above, and indicated in the methods section, the methods for selecting houses were dependent on a list provided by Yucatan MOH on premises of symptomatic ABV cases reported to the healthcare system. Unfortunately, we could not randomize or create other balancing algorithms; we just visited houses that were provided in the list for having a case within 1 month of receiving the information. Given *Ae. aegypti* seldom disperses beyond 100m, we saw no need in creating a distance threshold to select houses, which were distributed throughout Merida. We just aimed at getting as many houses as possible and as rapidly as possible to achieve our target of 200 houses. Our focus on houses with history of infection was based on the goals of the study, which are now more clearly stated. We see caveats in selecting houses with no apparent presence of infection. Most ABV infections are asymptomatic and selecting a ‘negative’ house without conducting PCR on all participants would lead us to the wrong assumption that no infection was present in the human population.**

*b.    I am unclear how the authors can justify that they are quantifying absolute vs. relative abundance of mosquitoes nor why this is an important distinction to make for the questions they are interested in addressing. To me relative abundance is absolute abundance (true abundance) multiplied by some estimate of capture efficiency. One can estimate absolute abundance by adjusting their measures of relative abundance by some imperfect detection rate. This has not been done in this study as currently written. In the current calculation of absolute abundance (sum aegypti over all of the 10 min intervals spanning 3 hr), the authors are assuming they have captured all of the aegypti in a given household. To me, this is just another measure of relative abundance (albeit likely a better one than just one 10 min interval) and if the authors want to get at absolute abundance than they need to adjust relative abundance by some metric of capture efficiency (which will go down with increasing effort). The authors may be able to get at this through the relationship between capture rate and time. If the authors do not want to do this, then they need to make their assumption that they have captured all of the aegypti in the house explicit in the manuscript and also discuss the implications for the interpretation of their results if this assumption is not upheld.*
**R. The justification for using absolute vs relative abundance is quite simple. Our previous paper (Koyoc‑Cardena et al. Parasites and Vectors 2019 12:250) shows that total catch is statistically undistinguishable from the estimated absolute abundance (see Fig. 3 in article). Therefore, we can conclude that what we call ‘total catch’ is an accurate representation of the absolute abundance of Ae. aegypti at the time of collection. A 10-min collection is a sample of that total catch. While we appreciate some of the suggestions the reviewer outlines, we consider they are not required given our reported association between indices provided in the prior publication. We now include the following sentence in the methods section L235 “Total catch is an accurate estimate of *Ae. aegypti* absolute density indoors, as our prior study showed that both measures did not differ statistically from each other”**

***c.    Statistical analyses:*** *I imagine that the ability to detect arbovirus infection in mosquitoes will not only depend on mosquito densities within the house but also the number of household members that were symptomatic. Do you have this information to include in your model analysis? Also, all of the models constructed, and the associated AIC scores should be included somewhere in the manuscript along with other metrics of model performance (residual analysis, etc.). Finally, I am not an expert on Bayesian approaches, but maybe these approaches would be appropriate for these data that explore, given a conditional probability that the household has arbovirus, the probability of detecting that arbovirus in mosquitoes and what factors (e.g. sampling effort and mosquito density) influence that probability?*

**R. We strongly disagree with the reviewer. The rate of infection would not depend on the number of symptomatic individuals, but on the number of infectious individuals (including visitors). Symptomatic cases are small fraction of all cases, and evidence from other studies indicates they are a poor predictor of ABV transmission risk. Also, we have deleted the sentence about AIC, as we did not perform model selection in this study. We just ran GLMMs to test simple statistical hypothesis of association between infection and specific variables (sella, total catch, EIR, VC). We also disagree in the recommendation of the use of Bayesian inference without a clear justification of why. Our models include random intercepts to capture the individual variability inherent of each house, and we consider no further refinements of the model are justified. One may be able to fit a more complex model, but its utility of value is not justified.**

***d.    Estimates of risk:*** *Why use both EIR and VC? For the questions being addressed in this study as well as the implications for using data like these for targeted control, EIR seems a more relevant metric of risk. Further, the parameters comprising EIR can be directly estimated from the data generated in this study vs. VC, which has parameters that this study did not directly measure and likely change based on environmental conditions (mosquito survival rate and the extrinsic incubation period). Also, why is the probability of a mosquito becoming infected after biting an infectious person not included? This formulation of VC also has dimension issues. See: Massad Eduardo, Coutinho Francisco Antonio Bezerra. Vectorial capacity, basic reproduction number, force of infection and all that: formal notation to complete and adjust their classical concepts and equations. Mem. Inst. Oswaldo Cruz. 2012;  107 (4): 564-567. [https://nam03.safelinks.protection.outlook.com/?url=https%3A%2F%2Fdoi.org%2F10.1590%2FS0074-02762012000400022&amp;data=02%7C01%7Cgmvazqu%40emory.edu%7C3d6c1d6df2674e5f799108d859f93c41%7Ce004fb9cb0a4424fbcd0322606d5df38%7C0%7C0%7C637358274596969966&amp;sdata=Z5QgjnqXjffHPERO7F837o0MV3%2FRsWPzY53G%2FoNndYA%3D&amp;reserved=0](https://nam03.safelinks.protection.outlook.com/?url=https%3A%2F%2Fdoi.org%2F10.1590%2FS0074-02762012000400022&data=02%7C01%7Codkirstein%40emory.edu%7C956130ae7a2f4fd49e6d08d85a5d11e3%7Ce004fb9cb0a4424fbcd0322606d5df38%7C0%7C0%7C637358703064956955&sdata=rBw5YtluG6QHeuacF7MUe3rTI4n9M6m%2BHOOgj8iZgpE%3D&reserved=0).*

*I would recommend dropping VC unless further justification is provided and the potential limitations of VC are acknowledged in the discussion section.*
**R. VC is included because it is a metric that has been calculated for ABVs, and because our goal was to associate vector density with key entomological metrics such as EIR and VC. The formulation we have chosen is the classic for VC, and we disagree with the suggestion of utilizing Massad’s approach because we are using a dt=1 day, which turns their formulation into the classic equation of VC. This old publication (2012) has not been addressed by recent and more advance studies utilizing VC. Our own self-criticism is that the VC equation did not include a term to account for heterogeneous biting. But, as explained to reviewer 1, given we were interested in the sensitivity of VC ad EIR to sample versus total catch, the impact of heterogeneous biting would apply to both calculations. After all, many studies have been published recently using VC in its current form. Here, we caution how such studies may vary if considering the sample vs making adjustments to account for Ae. aegypti absolute density.**

***3.    Discussion:***
*a.    In the introduction and the discussion section, the authors make statements about the sensitivity of the Prokopack aspirators in detecting arbovirus infected mosquitoes and how this probability detection will be dependent on collection method. I would make sure that the language here is careful and reflects what the study actually examined. As currently reads, it seems as if the authors are arguing that the Prokopack is more sensitive to other collection methods (CDC backpack aspirator and other trapping methods). While this might be true, this is not what the study actually set out to measure. So clarify the language throughout to reflect how the factors measured (e.g. mosquito density, amount of effort, number of blood-fed mosquitoes, etc.) affect the sensitivity of detecting arbovirus infected mosquitoes.*
**R. We never made any statement claiming Prokopacks are more or less sensitive than other methods. We simply stated that our approach for calibrating Prokopacks (which may be transferable to other adult aspiration devices) has not been attempted with other methods such as traps. In reviewing the text we made sure no confusing claims remained, and included the following sentence: “Furthermore, our findings may be transferable to other adult aspiration devices that operate under the same procedures and assumptions as the Prokopack (e.g., CDC back-pack aspirator,”**

*b.    I think there are alternative (but not mutually exclusive) hypotheses that explain the overdispersion of infected mosquitoes across houses sampled. The aggregation of mosquito bites on a few attractive hosts could potentially explain this, but you are then assuming that mosquitoes can actively select certain households. As mentioned by the authors, aegypti does not disperse very far, so depending on how the sampled households in this study are located across space, there may be other explanations for the overdispersion. For example, variation in housing across the city could affect the accessibility of indoor habitat for questing and resting females, which would then affect the overall mosquito densities in a house. Further, what factors drive spatial variation in human arbovirus infections? Where the relatively few households that generated the most arbovirus infections in mosquitoes connected in any way or located spatially in a similar area? A broader discussion on potential mechanisms here is warranted.*
**R. We agree multiple factors can explain overdispersion of mosquitoes. We have expanded this component in the discussion section.**

*c.    I would add a concluding paragraph that summarizes the key implications the authors would like the readers to walk away with*
**R. We included this in the first paragraph of the discussion.**

***Methods***

*a.    More information is needed in the study design. How were specific houses identified for sampling? Was it solely based on infection status of household members or did the authors try to randomize across households that had a history of symptomatic infection? Also, why only focus on households that had a history of symptomatic infection (this might become clearer when the study questions are added)? How were these homes distributed across space and time? Did you select homes to control for inter-home correlation by choosing homes that were spaced a certain distance apart? How many times were homes sampled (once vs. repeatedly)? I realize that these methods are likely associated with the reference provided in the text, but it would be nice to have some of this here to contextualize the discussion of the results.*

**R. This comment is a duplicate of the one we already addressed in the ‘General Comments’ section.**  *b.    I am unclear how the authors can justify that they are quantifying absolute vs. relative abundance of mosquitoes nor why this is an important distinction to make for the questions they are interested in addressing. To me relative abundance is absolute abundance (true abundance) multiplied by some estimate of capture efficiency. One can estimate absolute abundance by adjusting their measures of relative abundance by some imperfect detection rate. This has not been done in this study as currently written. In the current calculation of absolute abundance (sum aegypti over all of the 10 min intervals spanning 3 hr), the authors are assuming they have captured all of the aegypti in a given household. To me, this is just another measure of relative abundance (albeit likely a better one than just one 10 min interval) and if the authors want to get at absolute abundance than they need to adjust relative abundance by some metric of capture efficiency (which will go down with
increasing effort). The authors may be able to get at this through the relationship between capture rate and time. If the authors do not want to do this, then they need to make their assumption that they have captured all of the aegypti in the house explicit in the manuscript and also discuss the implications for the interpretation of their results if this assumption is not upheld.*
**R. This comment is a duplicate of the one we already addressed in the ‘General Comments’ section.**

*c.    Statistical analyses: I imagine that the ability to detect arbovirus infection in mosquitoes will not only depend on mosquito densities within the house but also the number of household members that were symptomatic. Do you have this information to include in your model analysis? Also, all of the models constructed, and the associated AIC scores should be included somewhere in the manuscript along with other metrics of model performance (residual analysis, etc.). Finally, I am not an expert on Bayesian approaches, but maybe these approaches would be appropriate for these data that explore, given a conditional probability that the household has arbovirus, the probability of detecting that arbovirus in mosquitoes and what factors (e.g. sampling effort and mosquito density) influence that probability?*
**R. This comment is a duplicate of the one we already addressed in the ‘General Comments’ section.**

*d.    Estimates of risk: Why use both EIR and VC? For the questions being addressed in this study as well as the implications for using data like these for targeted control, EIR seems a more relevant metric of risk. Further, the parameters comprising EIR can be directly estimated from the data generated in this study vs. VC, which has parameters that this study did not directly measure and likely change based on environmental conditions (mosquito survival rate and the extrinsic incubation period). Also, why is the probability of a mosquito becoming infected after biting an infectious person not included? This formulation of VC also has dimension issues. See: Massad Eduardo, Coutinho Francisco Antonio Bezerra. Vectorial capacity, basic reproduction number, force of infection and all that: formal notation to complete and adjust their classical concepts and equations. Mem. Inst. Oswaldo Cruz. 2012;  107 (4): 564 - 567.*

[*https://nam03.safelinks.protection.outlook.com/?url=https%3A%2F%2Fdoi.org%2F10.1590%2FS0074-02762012000400022&amp;data=02%7C01%7Cgmvazqu%40emory.edu%7C3d6c1d6df2674e5f799108d859f93c41%7Ce004fb9cb0a4424fbcd0322606d5df38%7C0%7C0%7C637358274596969966&amp;sdata=Z5QgjnqXjffHPERO7F837o0MV3%2FRsWPzY53G%2FoNndYA%3D&amp;reserved=0*](https://nam03.safelinks.protection.outlook.com/?url=https%3A%2F%2Fdoi.org%2F10.1590%2FS0074-02762012000400022&amp;data=02%7C01%7Cgmvazqu%40emory.edu%7C3d6c1d6df2674e5f799108d859f93c41%7Ce004fb9cb0a4424fbcd0322606d5df38%7C0%7C0%7C637358274596969966&amp;sdata=Z5QgjnqXjffHPERO7F837o0MV3%2FRsWPzY53G%2FoNndYA%3D&amp;reserved=0)*.*

*I would recommend dropping VC unless further justification is provided and the potential limitations of VC are acknowledged in the discussion section.*
**R. This comment is a duplicate of the one we already addressed in the ‘General Comments’ section.**

***Results***

*Maybe? The questions of the study need to be clearly outlined along with the associated response variables. The tables and figures need work largely in clarifying the legends and some of the axes. These are noted in the uploaded document.*
**R. We have addressed all the comments and responded all the queries of the reviewer.**

***Conclusions***

*The conclusions are mostly supported by the data presented, but I did note when this was not the case throughout the discussion in the uploaded document. The limitations of the analysis are not currently described. The authors should include a paragraph in the discussion addressing study limitations, assumptions made, and implications for interpretation of their results. Public health relevance is addressed, but I have made suggestions on how to clarify this in the discussion.*

**R. We now include limitations both in a paragraph and in different sections of the discussion.**

***Editorial and Data Presentation Modifications?***

*Minor Concerns:*

*Line 39-40: Ae. aegypti needs to be italicized*

**R. Done**

*Line 87-91: consider combining these two sentences with the following paragraph. Generally, for a paragraph to stand alone, there needs to be a minimum of three sentences. I also think these two sentences thematically align with the information in the following paragraph.*

**R. Done.**

*Line 114: “was detected 10 days...” in what? I am assuming field collected mosquitoes?*

**R. Yes. Added.**

*Line 148: (within 1 month) should be (within one month) – generally all numbers from 1-9 should be written out unless at the beginning of a sentence. I would go through the entire manuscript for this one, as there are multiple instances where this needs to be corrected.*

**R. Done.**

*Line 234: Based on the topic sentence of this paragraph (lines 231-232), VC and its definition should follow EIR and its definition before getting into the specifics of each metric.*

**R. Considered and accepted.**

*Line 244: n = extrinsic incubation period should be the number of days on average it takes for the mosquito to become infectious (so, I am assuming this is 5 days). As currently written, this is defined as the extrinsic incubation rate (1/5 days), which would be incorrect.*

**R. That is correct. EIP = 5, and we expressed it within the formulation of n, which is equal to 1/EIP. We clarified this in the text.**

*Line 257-258: “Of the total ABV-infected females, 38 (22.9%) had evidence of infection in their heads; 33 (86.8%) of them were positive for CHIKV...” Do the authors mean 33 of the total ABV-infected females or 33 of the* *ABV-infected females with positive heads?*

**R. We refer to 33 of the ABV-infected females with positive heads. The statement was addressed and clarified.**

*In general, the authors should make it really clear how the percentages presented throughout this manuscript are calculated. Especially in the associated tables (1-3), which should stand alone from the text, it is completely unclear what the percentages in parentheses are referring to.*

**R. We now indicate in the table what those %s are referring to.**

*Line 272: “follow” should read “follows”*

**R. Done.**

*Line 281: “When mosquito density was high…” I would clarify that this is “**When within household mosquito density was high…”*

**R. Considered and accepted.**

*Line 340: “considered a gold standard for indoor adult Ae. aegypti collections” – While this might be the case, I do not think this statement fits with what this sentence is trying to convey and I would recommend removing it. Especially since this study is not comparing sensitivity across different collection methods, this is confusing.*

**R. We rewrote the first paragraph and have removed that statement.**

*Line 345: “will be a function of the collection method used” – I would provide a reference here to support this statement as this study did not actually measure this.*

**R. We now add reference to Silver’s ‘Mosquito Ecology’ book, that provides a complete reference to sampling mosquito methods and their issues. Also, Scott and Morrison touch on that.**

*Line 348-350: “These data show that the low apparent…may also be a function of the sensitivity of the collection method.” I would clarify this sentence to reflect what this study actually measured – again, this study did not demonstrate the effectiveness of the Prokopack method relative to other collection methods, so starting the sentence with “these data show…” is somewhat misleading.*

**R. Point taken. We toned down the statement.**

*Line 355-357: “we evidenced its powerful epidemiological effect in the strong overdispersion of infection in collected mosquitoes.” I am not sure what the authors really are saying here. Please clarify.*

**R. We have clarified this paragraph, upon request from another reviewer, and that statement is not excluded in this version. We now include the following sentence: “Multiple mechanisms could have been responsible of such overdispersion, including aggregation of bites on one or a few infected individuals, mosquito biting on multiple viremic visitors to the house, or the dispersal of infected mosquitoes from nearby premises.”.**

*Also, can you really say that your data demonstrate heterogeneous biting? Or rather, that some households had more mosquitoes than others and the distribution of households with high infection rates also had high mosquito densities, so something to do with variation in housing infrastructure and mosquito habitat? I agree that people are not equally attractive, but I think other factors are likely driving the distribution of infected mosquitoes.*

**R. We are not implying that we “demonstrated heterogenous biting”, in fact we mention how should have been measured. We are discussing an obvious possible situation which is implied from our data.**

*Line 358-359: see above comment. Also, do you know how many members of each household sampled were symptomatic for arbovirus infection?*

**R. See explanations above for Reviewer #2. Unfortunately, we do not. We mention this as a limitation.**

*Line 369: this sentence is not as clear as it could be. I suggest: “Wolbachia population replacement or suppression approaches rely on the release of* *Wolbachia infected adults,* *which when mated to wildtype,* *uninfected adults render the population incompetent to transmission or reduce adult female density, respectively.”*

**R. Considered and accepted.**

*Line 372-376: This sentence needs clarification and this paragraph needs a better topic statement - I think I know what you are trying to say here...that the effective deployment and assessment of these technologies require good estimates of mosquito distributions across households, within household mosquito densities, and arbovirus infection rates. You should lead with this and then provide examples demonstrating why this is important.*

**R. We expanded this sentence.**  *Line 389: “bias” should be “to bias”*
**R. Done**

*Line 393-396: The study design of the referenced study was different from the one in this study. It appears from the referenced study that they did not target efforts to households with confirmed symptomatic infection in household members. So, the prevalence would naturally be much lower than this study I would expect. So, I don’t understand the point the authors are trying to make here.*

**R. Based on feedback from Reviewer 2, we edited this paragraph. We focus on the low dengue infection rates, rather than comparison between studies. We added other references that support those findings.**

*Line 406: “This data aligns” should be “These data align”*
**R. Done**

*Line 413: “thought” should be “**through”*

**R. Done**

*Line 418-419: “DENV-4 the predominated in the most years” is awkward. Consider “This result lines up with previous results obtained from different work in the area where all four DENV serotypes were found circulating in Merida**, with DENV-4 being the predominant serotype in most years along with DENV-1 and DENV-3 serotypes.”*
**R. Considered and accepted.**

*Line 424-426: Well...there is the salivary gland barrier that needs to be breached and can be a bottleneck in the infection process...so just because you see it in the head does not mean it is in the saliva...I would be careful here - maybe acknowledge this and then state that it is likely that these mosquitoes are infectious.*

**R. Considered and clarified in the text**

*Line 435: I would argue that VC in this sense is not as good as EIR because multiple parameters you assume values for, and these will be sensitive to environmental conditions.*
**R. We disagree and prefer to leave the reader to decide if your statement is true. VC is often calculated for ABVs. Here we bring to the attention how changes between absolute and relative vector density indoors as well as mosquito infection influence VC and EIR. So, our aim of keeping them both is justified and also has not received any criticism by any of the other reviewers.**

*Table 1: from the table legend, it is unclear how the percentages in the parentheses are calculated.*
**R. we added a reference to that.**

*Table 2: figure legend “either” ??? remove? Also, the are the percentages in this table reflecting the number of mosquitoes with positive heads out of total number of mosquitoes infected. Captured?*

**R. Figure legend corrected.**

*Table 3: again, how are the percentages calculated – tables and figures need to stand alone. I am assuming these are the number of mosquitoes with positive heads divided by the number of mosquitoes captured with each Sella score?*
**R. corrected.**  *Figure 2 – maintain the same colors for each virus across each panel A and B – also line 697, 3 should be three*

**R. Done** *Figure 3 – the y-axis is confusing…it reads like the number, when this is really a probability of detection of infected females*
**R. Y-axis now reads “Probability of infection”**

*Figure 4 – what is the difference between A and B?*
**R. One is for individual houses (A) and the other for individual female mosquitoes (B)**

*Figure 5 – sample vs. total – do you mean first 10 min interval vs. the sum total across all 10 min intervals?*

**R. Yes. We call the latter ‘total catch’.**
